# Contribution of the bitter taste signaling pathway to lung inflammation during *Staphylococcus aureus*-induced pneumonia

**DOI:** 10.3389/fimmu.2025.1647780

**Published:** 2025-10-09

**Authors:** Ling-Ling Liu, Feng Li, Meng-Min Zhu, Bo-Wen Niu, Yu Huang, Lixiang Chen, Hua Yang, Boyin Qin, Xiaohui Zhou

**Affiliations:** ^1^ Department of Laboratory Animal Science, Shanghai Public Health Clinical Center, Fudan Univeristy, Shanghai, China; ^2^ Department of Biology, College of Life Sciences, Shanghai Normal University, Shanghai, China

**Keywords:** bitter taste receptor, *Staphylococcus aureus*, pneumonia, inflammation, cytokines

## Abstract

Bitter taste receptors (TAS2Rs), initially identified for chemosensory roles in the tongue, are expressed in extraoral tissues, including the airways. However, to date, it remains unclear whether bitter signaling is associated with susceptibility to bacterial infection in the lower airways and whether bitter signaling actually participates in the immune response in lung infection has yet to be genetically established. Here, we investigated the role of TAS2R signaling in *Staphylococcus aureus*-induced murine pneumonia via wild-type (WT) and several mutants (mTas2r104^-/-^/105^-/-^, mTas2r105^-/-^/114^-/-^, mTas2r104^-/-^/105^-/-^/114^-/-^, Gnat3^-/-^ and Gnat3^-/–^mTas2r104^-/-^/105^-/-^) mice. Genetic disruption of TAS2Rs altered compensatory expression of other bitter receptors in the trachea and lungs, but did not affect immune cell composition in the lungs or thymus. Bitter receptor-deficient mice exhibited exacerbated pulmonary lesions at day 3 (D3) post-infection. Pulmonary infection significantly upregulated *mTas2r105*,*106*, *107*, *108*, *126*, *136*, *138* and *Gnat3* in the lung. TAS2R signaling deficiency downregulated the expression of cytokines (e.g., IL-10, MIP-2) and antimicrobial peptides in the lungs and trachea, increased CD68+ macrophages in D3 lung tissues, amplified Ki67+ cell proliferation in alveolar and bronchiolar regions, and even impaired recovery from lung injury by day 14 (D14). Mechanistically, bitter taste pathway disruption dysregulated the mTOR pathway, reduced eNOS expression, and delayed resolution of pneumonia-induced injury. In summary, the current results collectively indicate that bitter taste signaling can modulate innate immune and inflammatory responses during S. aureus-induced lung infection.

## 1 Introduction

Infectious diseases remain a global public health threat, affecting both developed and resource-limited settings. *Staphylococcus aureus* exemplifies this challenge, causing pathologies ranging from benign skin infections to life-threatening conditions such as endocarditis and pneumonia (1). Notably, S. aureus accounts for 25% of community-acquired pneumonia cases and nearly 50% of hospital-acquired pneumonia cases, driving high morbidity and mortality in immunocompromised ICU patients and young individuals with cystic fibrosis (2). The deeper insights into the pathophysiology of *S. aureus* infections are urgently needed.

Pulmonary innate defense against *S. aureus* relies on epithelial cells and phagocytes, including alveolar macrophages and neutrophils. Dysregulation of these cells can exacerbate pneumonia progression (3–7). Beyond their canonical role in taste perception, bitter taste receptors (TAS2Rs), a class of G protein-coupled receptors, mediate diverse extraoral functions via interactions with G proteins, effector enzymes (e.g., phospholipase Cβ2), and second messengers (Ca^2+^, cAMP) (8–11). In the respiratory system, TAS2Rs are expressed in the human bronchi (12–14), airway epithelial cells (15, 16), immune cells, including alveolar macrophages (17, 18), neutrophils (19,) mast cells (20) and blood leukocytes (21). These receptors regulate critical processes, including innate immunity, mucociliary clearance, and inflammation (14, 22, 23). However, to date, it remains unclear whether bitter signaling is associated with susceptibility to bacterial infection in the lower airways and whether bitter signaling actually participates in the immune response in lung infection.

Here, we investigate the contribution of bitter taste signaling to *S. aureus*-induced pneumonia using wild-type (WT) and TAS2R-deficient mice. We demonstrate that genetic disruption of bitter signaling attenuates pulmonary cytokine and antimicrobial peptide (AMP) production, exacerbates histopathological damage, and delays recovery from infection. Mechanistically, TAS2R deficiency leads to the dysfunction of mTOR pathway, suppresses endothelial nitric oxide synthase (eNOS) expression, and impairs lung injury repair. These findings establish a previously unrecognized role for bitter taste receptors in modulating innate immune responses during bacterial pneumonia.

## 2 Materials and methods

### 2.1 Mice

Mice aged 8–12 weeks were housed in the SPF animal facility of the Experimental Animal Department at the Shanghai Public Health Clinical Center. Bitter taste receptor-deficient mice (mTas2r104^-/-^/105^-/-^, mTas2r105^-/-^/114^-/-^, mTas2r104^-/-^/105^-/-^/114^-/-^, Gnat3^-/-^, Gnat3-Tas2r104^-/-^/105^-/-^) on the C57BL/6 background were generated as previously described (10). Pathogen-free C57BL/6 mice were purchased from Shanghai Laboratory Animal Center (SLAC, Shanghai, China). All animal experiments were approved by the Animal Care and Use Committee of Shanghai Public Health Clinical Center (Shanghai, China).

### 2.2 Bacterial growth and infection

The frozen *S. aureus* Newman strain was thawed and inoculated onto a mannitol agar plate by streaking and allowed to grow overnight at 37 °C. After picked, *S. aureus was grown* Trypto-casein soja broth (TSB BD Biosciences) at 37 °C with shaking (200 rpm) until the log phase. The bacteria were subsequently centrifuged twice and adjusted to a suspension of 5 ×10^10^ bacteria/ml. After anesthetized with isoflurane, these mice were infected intranasally with 50 μl (10^9^ CFU) bacteria, and maintained in the Animal Biosafety Level II laboratory.

### 2.3 Bacterial load in the lungs

The infected lung tissue was excised, weighed and homogenized in sterile PBS at a ratio of 1 ml of PBS per gram of tissue. After 10-fold dilutions. the samples were then plated and incubated at 37 °C for 48 hours. Bacterial load was obtained by counting colony (7).

### 2.4 RNA extraction and qRT-PCR

An RNA extraction kit (19211ES60, Yisheng, China) was used to extract and purify the total RNA of the mouse lung and tracheal tissues. The above mouse tissue RNA was reverse transcribed at 42 °C for 30 min via a reverse transcription kit (1123ES60, Yisheng, China). The results of the qPCR experiments were analyzed via a SYBR Green Master Mix kit (11201ES80, Yisheng, China) and a quantitative PCR instrument (FQD-96A, Bioer, China), and the sequences of the primers used and other relevant information are listed in **Supplementary Table S1**.

### 2.5 Western blotting

The total protein was extracted and quantified from the lung samples as previous reports (24). In brief, equal amounts of protein samples were separated by SDS-PAGE and transferred to PVDF membranes (ISEQ00010, Merck Millipore, UK). The PVDF membranes were then blocked and incubated with primary antibodies overnight at 4 °C (**Supplementary Table S2**). After washed and incubated with the corresponding HRP-conjugated secondary antibodies (HS101-01, TransGen, China) for 2 h, immunoreactive bands were detected with an enhanced chemiluminescence detection reagent. Band intensities were quantified via ImageJ software, and the results were normalized to β-actin levels and reported as intensities relative to those of the controls.

### 2.6 Immunofluorescence staining

Lung samples were fixed overnight in 4% paraformaldehyde, cryosectioned and subjected to immunofluorescence staining, as previous reports (24). After being permeabilized in 0.3% TX-100 and blocked in 5% bovine serum albumin (BSA), the sections were incubated with primary antibodies (**Supplementary Table S2**) at 4°C overnight. The sections were then incubated with the secondary antibody Alexa-488-conjugated mouse anti-rabbit (Invitrogen, A-21202, 1:800) for 1 h at room temperature and subsequently incubated with 4’,6-diamidino-2-phenylindole (DAPI) for 0.5 h. All images were collected with a confocal microscope (Leica TCS SP5).

### 2.7 Histopathology

Lung tissues were fixed in 4% paraformaldehyde, embedded in paraffin, and sectioned at a thickness of 5 μm. Lung histopathological injury was assessed by hematoxylin and eosin (H&E) staining. Entire lung sections stained with H&E were automatically were captured under an artificial pathology section scanner (KF-PRO-120, KFBIO). At least 5 points each slides were assessed by a histopathologic inflammatory scoring system as described previously in a hamster M. pneumoniae infection model (25). This scoring system has been previously used in other mouse models of respiratory infections (7, 26, 27). Briefly, a final score per mouse on a scale of 0 to 26 was obtained by assessing the quantity and quality of peribronchiolar and peribronchial inflammatory infiltrates, luminal exudates, perivascular infiltrates, and parenchymal pneumonia. A. Peribronchial/peribronchiolar infiltration: 0 = no infiltration; 1 = minimal infiltration (<25%); 2 = moderate infiltration (25%~75%); 3 = complete infiltration (>75%). B. Qualitative assessment of peribronchial/peribronchiolar infiltration: 0 = no infiltration; 1 = mild infiltration; 2 = moderate infiltration; 3 = severe infiltration. C. Bronchial/bronchiolar lumen dissection: 0 = no exudation; 1 = mild exudation (<25%); 2 = severe exudation (>25%). D. Perivascular infiltration: 0 = no infiltration; 1 = minimal infiltration (<10%); 2 = moderate infiltration (10%); 3 = extensive infiltration (>50%). E. Parenchymal pneumonia: 0 = no pneumonia; 3 = mild pneumonia; 5 = severe pneumonia. Total score = A + 3(B + C) + D + E. The scores for all images of each lung section were then averaged, and the composite score for that animal’s lung was considered as 1 data point for statistical analysis and individual data point in graphs.

### 2.8 Flow cytometry

A single-cell suspension was obtained from lungs and thymus as previous describe (7). The cells were then stained with the following antibodies. Zombie Aqua™ Fixable Viability Kit (Biolegend), APC/Cyanine7-conjugated anti-CD45 (clone 30-F11), FITC-conjugated anti-CD3 (clone 145-2C11), Pacific blue-conjugated anti-CD4 (clone GK1.5), Brilliant violet605-conjugated anti-CD8 (clone GK1.5), PE/Cyanine5-conjugated anti-CD19 (clone 6D5), PE-conjugated anti-CD11b (clone M1/70), and FITC-conjugated anti-LY6G (clone 1A8) antibodies were used. The data were analyzed via FlowJo v10.6.2.

### 2.9 Statistical analysis

SPSS 21.0 software was used to analyze significant differences. A homogeneity of variance test and normality test were performed for the experimental data. If multiple sets of variables were consistent with homogeneity of variance, analysis of variance (ANOVA) was used to compare multigroup variables, and the least significant difference (LSD) test was used to compare intergroup variables. If homogeneity of variance was not assumed, Dunnett’s T3 test was used to compare the intergroup variables. The values are presented as the means ± SDs. Values of *p* < 0.05 were considered significant.

## 3 Results:

### 3.1 mTas2r105 expression in trachea and mainstem bronchi

**Figure 1:**
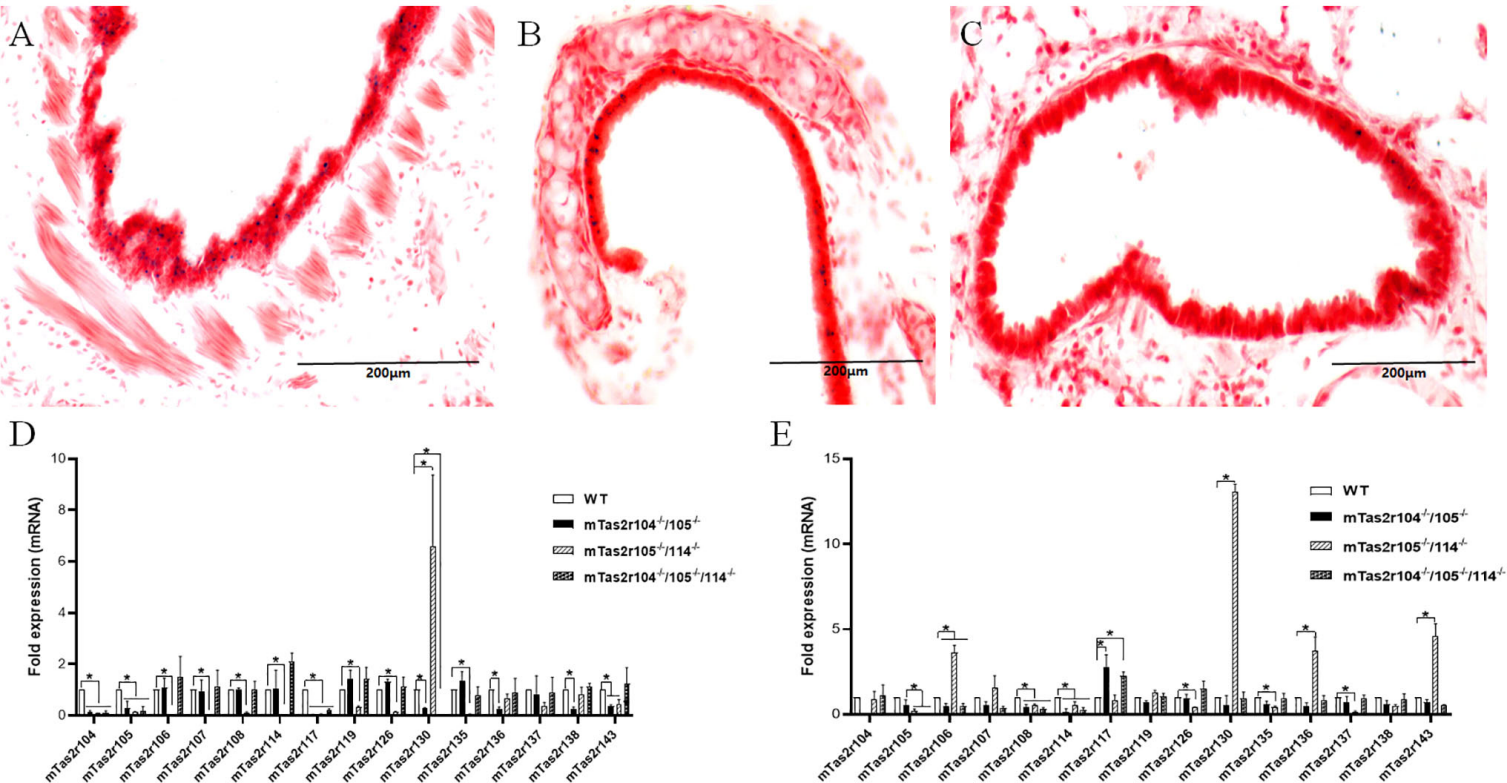
Expression of Tas2r105 in the respiratory tract. mTas2r105-Cre/GFP transgenic mice were crossed with R26:loxP-StoploxPLacZbpA transgenic mice. LacZ staining revealed the expression of Tas2r105 in the epithelium of the trachea **(A)** and primary bronchi **(B)** but rarely in the epithelium of the bronchioles **(C)**. In Tas2r104^-/-^/105^-/-^, Tas2r105^-/-^/114^-/-^, and Tas2r104^-/-^/105^-/-^/114^-/-^ mice, the expression of several mTas2rs, including Tas2r104, Tas2r105, Tas2r106, Tas2r114, Tas2r108, Tas2r119, Tas2r126, Tas2r135, Tas2r136, Tas2r138, and Tas2r143, in the trachea **(D)** and lung **(E)** was detected via qRT-PCR. The statistical significance of differences between WT and Tas2rs mice was assessed with a Student’s *t*-test. *P < 0.05. Scale bar 200 μm.

The airway has been used as a phenotypical tissue to explore the pathophysiology of extraoral TAS2Rs. In a previous study, we generated mTas2r105-Cre/GFP transgenic mice (28). This mouse line was crossed with R26:loxP-StoploxPLacZbpA transgenic mice and can be used to track the expression of the mTas2r105 gene cluster in extraoral tissues (28, 29). The frozen sections from the lung tissues of the double transgenic mice were stained for LacZ. LacZ-positive cells were observed in the epithelium of the trachea (**Figure 1A**) and primary bronchi (**Figure 1B**) but rarely in the epithelium of the bronchioles (**Figure 1C**). To further study the function of mTas2r105 in extraoral tissues, we generated several mTas2rs mutant mice by targeting mTas2105 gene clusters via the CRISPR/Cas9 technique (10). We profiled the expression of several mTas2rs highly expressed in large airways and the lungs (30, 31). In the trachea, the expression level of Tas2r117, mTas2r130 and was downregulated in mutant mice (**Figure 1D**). In the lungs, we observed upregulated expression of mTas2r106, 107, 130, 136, and 143 in mTas2r105^-/-^/114^-/-^ mice and downregulated expression of mTas2r108 in all mutant mice (**Figure 1E**). These changes indicated a compensatory or regulatory network among Tas2r genes, though the exact pathway remains unclear.

**Figure 2:**
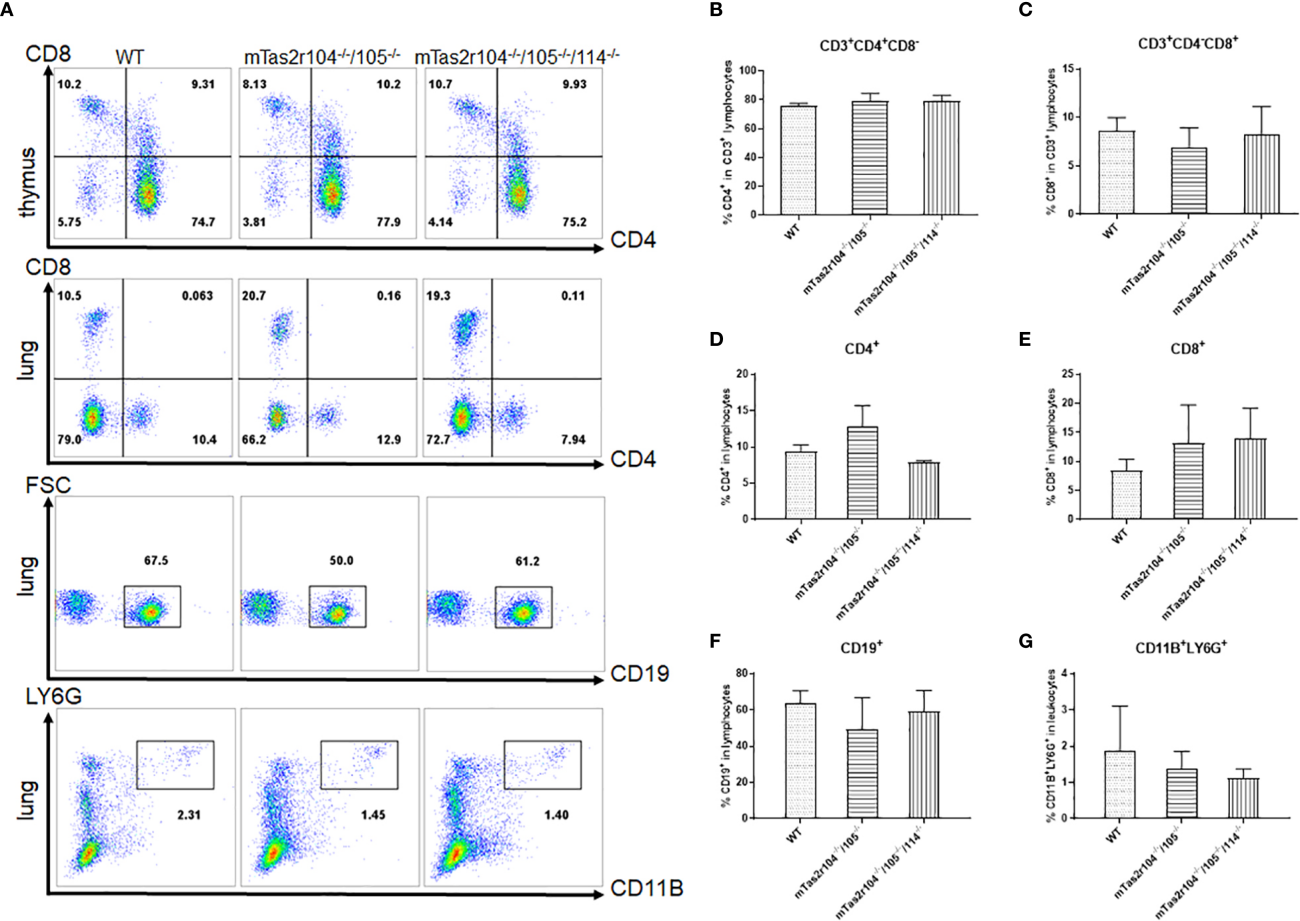
Immune cell subsets were analyzed by flow cytometry in the thymus and lungs of WT and mutant mice. **(A)** One representative FACS plot is presented, and the percentages indicate the proportions of immune cells in the thymus and lung (n = 4). The percentages of CD4+ **(B)** and CD8+ **(C)** T cells in the thymus. Percentages of CD4+ **(D)** and CD8+ **(E)** T cells in the lung. CD19+ B cells **(F)** in the lung. LY6G+ neutrophils **(G)** in the lung.

Previous studies have shown the expression of bitter taste receptors including mTas2r105, 108, 131 and 143 in the thymus (31, 32). We employed flow cytometry to investigate whether genetic mutations in bitter receptors affect the composition of lymphocytes in the lung and thymus (**Figure 2A**). The results revealed that the lymphocyte composition did not change in the thymus (**Figures 2B, C**) or lung (**Figures 2D–G**). Moreover, the number of neutrophils in the lung was also unchanged (**Figure 2G**), indicating that genetic mutation of the bitter receptor did not alter the composition of immune cells in the lung or thymus, and could not cause limited damage to T-cell development.

### 3.2 Impact of bitter receptor deficiency on bacterial load and cytokine production

To obtain insight into the role of bitter receptors in controlling infectious diseases caused by bacteria, we investigated the role of bitter receptors in a previously established murine model of *S aureus*-induced pneumonia (7). We compared the survival of wild-type (WT) and mutant mice (mTas2r104^-/-^/105^-/-^, mTas2r105^-/-^/114^-/-^, and mTas2r104^-/-^/105^-/-^/114^-/-^) after the intranasal instillation of 10^9^ bacteria per mouse (**Supplementary Figure S1A**). After 7 days, 90% of both groups of mice survived and recovered completely from the infection (**Supplementary Figure S1B**). Weight loss was more severe in mTas2r104/105 mice (**Supplementary Figure S1A**). Pulmonary infection significantly increased the mRNA level of IFNγ (**Supplementary Figure S2A**), TNFα (**Supplementary Figure S2B**) and IL-6 (**Supplementary Figure S2C**) at D1 in WT and mutant mice but not in mTas2r105^-/-^/114^-/-^ mice, indicating an impaired innate immune response in mTas2r105^-/-^/114^-/-^ mice.

QPCR analysis was further employed to investigate whether genetic mutation of bitter receptors affects the expression of virulence factors (**Supplementary Figures S2D-F**). Higher expression of the superantigens staphylococcal enterotoxin A (SEA) (**Supplementary Figure S2D**) and RNAIII (**Supplementary Figure S2F**) was observed only in mutant mice. We also observed a high expression level of 16 s at D1 in the lung homogenates from the WT and mutant mice (**Supplementary Figure S2E**). At D3, the SEA level returned to baseline (**Supplementary Figure S2D**), and a relatively high RNAIII level was still observed (**Supplementary Figure S2F**). Moreover, the bacterial load was further investigated in lung homogenates. Most CFUs were detected in mTas2r104^-/-^/105^-/-^ mice at D1 post-infection (**Supplementary Figure S2G**). These results indicate that bitter receptor signals may be involved in the regulation of virulence factors expression.

### 3.3 Bitter receptor-deficient mice display more severe pulmonary lesions at D3 after *S. aureus*-induced pneumonia

**Figure 3:**
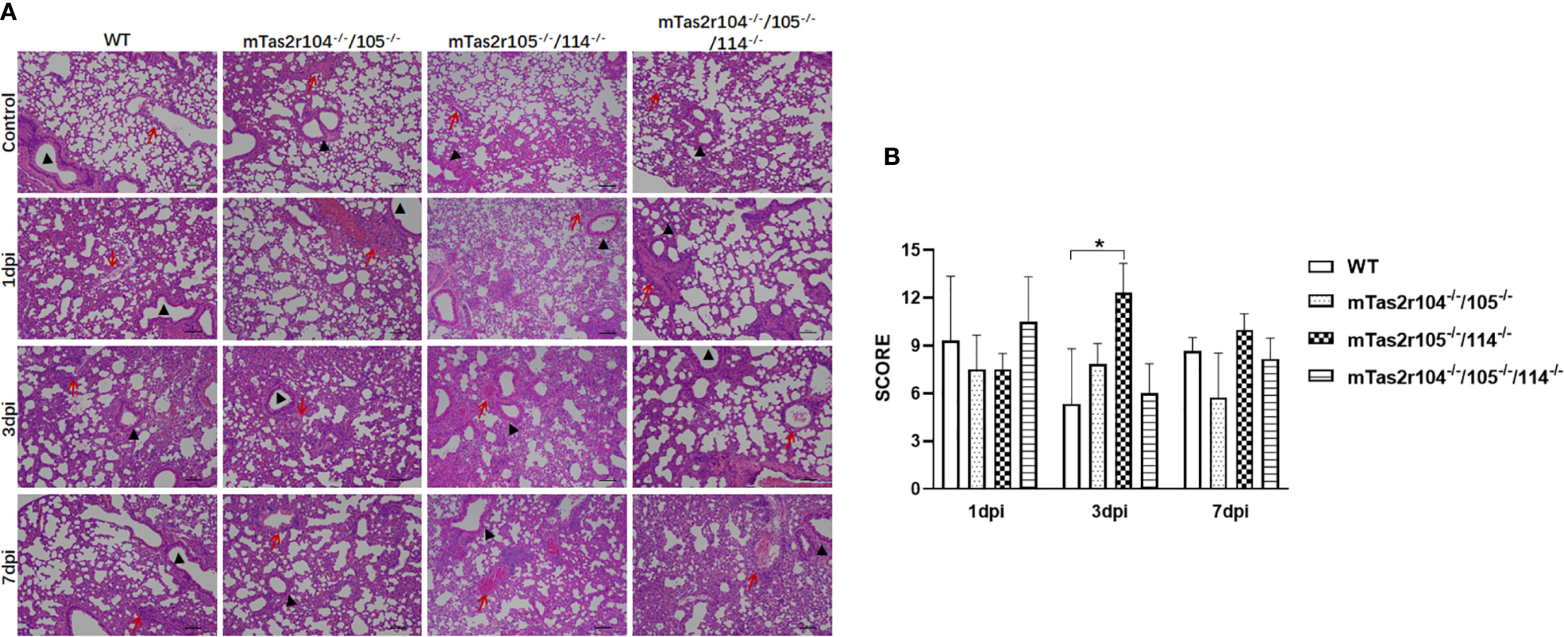
Histopathological examination of lung tissue from WT and mutant mice after intranasal exposure to *S. aureus* Newman. After intranasal exposure to *S. aureus* Newman, the lung tissues were harvested, prepared and stained with hematoxylin and eosin at the time points labeled in the figure. A representative histological image of the lungs of WT and mutant mice at the indicated time points is shown **(A)**. The whole lung section was scanned, and 6–8 regions from each sample were selected and scored according to the scoring system described in the Materials and Methods section **(B)**. Scale bar 100 μm.

To investigate the impact of *S. aureus* on the pulmonary parenchyma after intranasal inoculation, the mice were euthanized, and the lung histological analysis at D1 (early stage), D3 (middle stage) and D7 (late stage) was also performed. The different histopathological lesions at the three stages of infection are shown in **Figure 3A**. A histopathological scoring system was further used to assess the pathology of pulmonary infection (**Figure 3B**). A higher score was obtained at D3 in mTas2r105^-/-^/114^-/-^ mice, indicating that different pulmonary lesions may be observed among the three mutant strains after *S. aureus*-induced pneumonia.

### 3.4 Genetic mutation of the bitter receptor signaling pathway downregulates the expression of cytokines and antimicrobial peptides

The above results suggested that genetic mutation of bitter taste receptor aggravated pathological injury of lung. To further investigate the role of bitter taste signaling in *S. aureus*-induced pneumonia, we established a murine model of *S. aureus*-induced pneumonia in Gnat3^-/-^ and Gnat3^-/–^mTas2r104^-/-^/105^-/-^ mice, which can genetically mutate G protein α subunit (Gnat3) and is thought to eliminate most bitter taste signals (10, 11, 33). Similar to the previous situation, lower mortality was observed in these mutant mice. After 7 days, 80% of the WT and mutant mice survived and recovered completely from the infection (**Supplementary Figure S1B**). However, as shown in **Supplementary Figure 1C**, weight loss was significantly lower for mTas2r105^-/-^/114^-/-^, mTas2r104^-/-^/105^-/-^/114^-/-^, and Gnat3^-/-^ animals. In contrast, Gnat3^-/–^mTas2r104^-/-^/105^-/-^ mice and WT mice regained their initial weight on day 7 (**Supplementary Figure S1B**).

**Figure 4:**
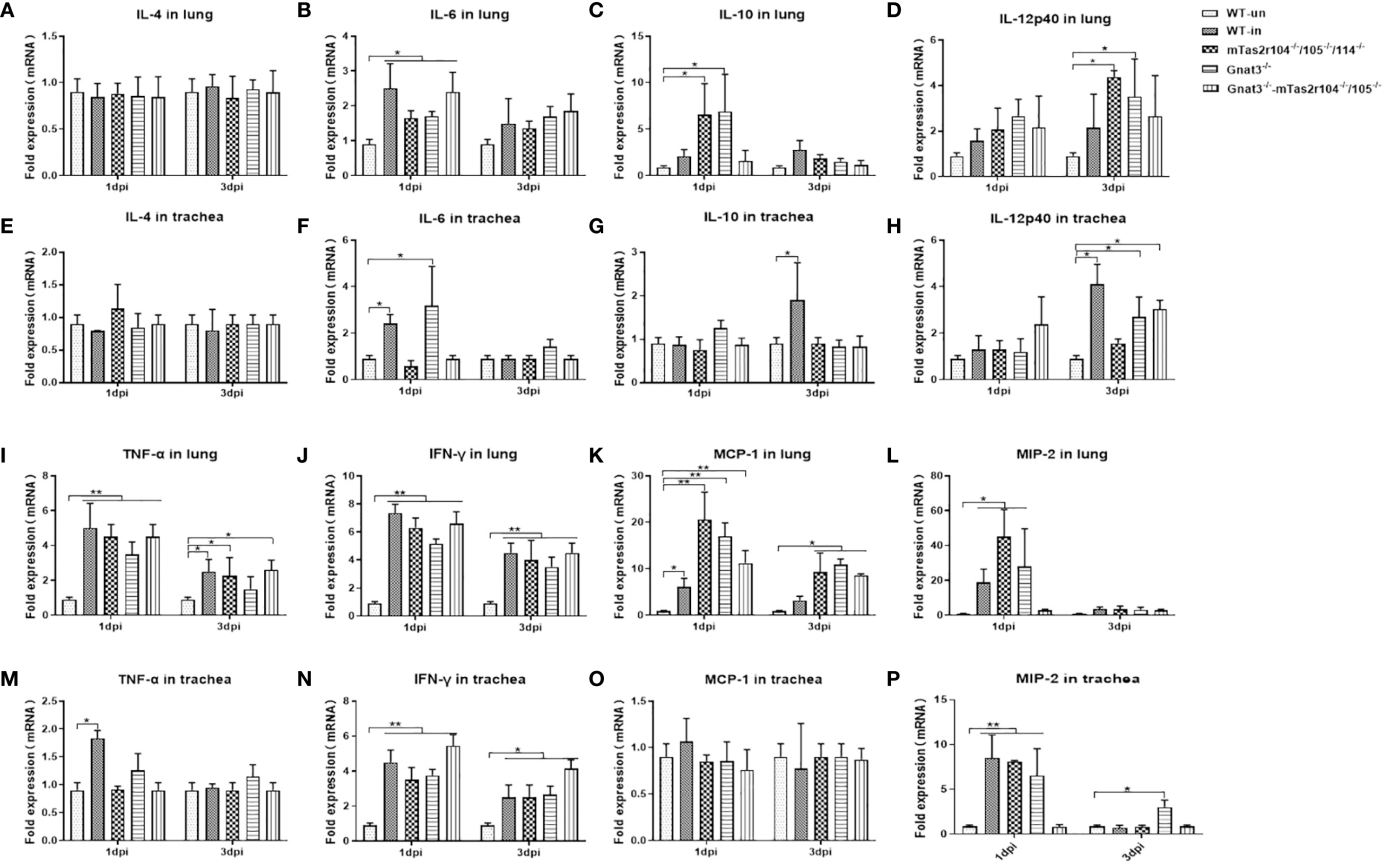
Bitter receptor signaling deficiency caused a decreased expression of cytokines after *S. aureus* Newman infection. Inflammatory cytokines kinetics in response to *S. aureus* Newman induced Pneumonia at D1 and D3 post-infection. Real-time PCR was used to analyze the expression levels of cytokines with specifically designed oligonucleotides. Cytokines are labeled in the histogram. **(A)** IL-4, **(B)** IL-6, **(C)** IL-10, **(D)** IL-12p40 in the lungs. **(E)** IL-4 **(F)** IL-6 **(G)** IL-10 **(H)** IL-12p40 in the trachea. **(I)** TNF-α, **(J)** IFN-γ, **(K)** MCP-1, **(L)** MIP-2 in the lungs. **(M)** TNF-α, **(N)** IFN-γ, **(O)** MCP-1, **(P)** MIP-2 in the trachea. The data are shown as the means ± SEMs (n = 3-5). The Dunnett test, or least significant difference (LSD) test, was used to analyze significant differences. *P < 0.05, **P < 0.01, ***P < 0.001, ****P < 0.0000.

The levels of immunoreactive cytokines and chemokines were measured in lung homogenates harvested 24 h and 72 h after inhalation of *S. aureus* (**Figures 4A-P**). Infection-related induction of most chemokines was evident in both WT and mutant mice, but responses were significantly blunted in Gnat3-mTas2r104^-/-^/105^-/-^ animals. The expression of IL-6 (**Figure 4B**), TNFα (**Figure 4I**) and IFNγ (**Figure 4J**) did not differ significantly between mutant and WT mice. A significant increase in MCP-1 expression was observed at D1 and D3 in the lungs of mutant mice (**Figure 4K**). More interestingly, the expression levels of IL-10 (**Figure 4C**) and MIP-2 (**Figure 4L**) were significantly increased in the lungs of mTas2r104^-/-^/105^-/-^/114^-/-^ and Gnat3^-/-^ mice, but no significant change, especially for MIP-2, was detected in the lungs and trachea of Gnat3^-/–^mTas2r104^-/-^/105^-/-^ mice, indicating that genetic mutation of the bitter receptor signaling pathway has a vital impact on the expression of MCP-1, IL-10 and MIP-2.

**Figure 5:**
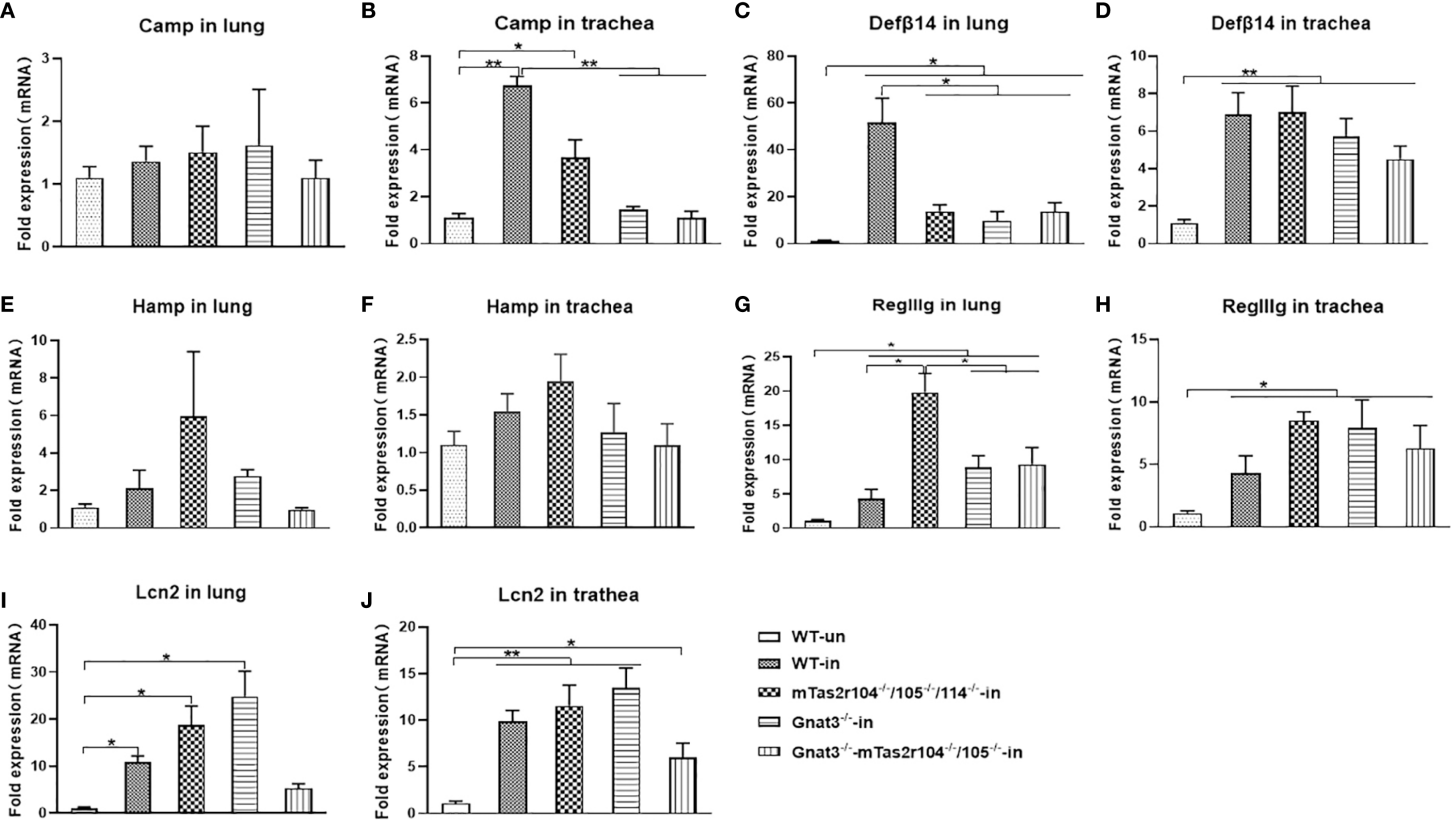
Bitter receptor signaling deficiency caused changes in the mRNA expression of antimicrobial peptides after the *S. aureus* Newman infection. The expression of antimicrobial peptides in *S. aureus* induced Pneumonia at D1 post-infection. Antimicrobial peptides are labeled in the histogram. **(A)** Camp in the lung. **(B)** Increased expression of Camp in the trachea of WT and Tas2r104^-/-^/105^-/-^/114^-/-^ mice. **(C)** DefA14 in the lung, the highest expression of DefA14 was detected in WT mice. **(D)** DefA14 in the trachea. **(E)** Hamp in the lung. **(F)** Hamp in trachea. **(G)** RegIIg in the lung. **(H)** RegIIg in the trachea. **(I)** Lcn2 in the lung. **(J)** Lcn2 in the trachea. The data are shown as the means ± SEMs (n = 3-5). The Dunnett test, or least significant difference (LSD) test, was used to analyze significant differences. *P < 0.05, **P < 0.01, ***P < 0.001, ****P < 0.0000.

We also investigated the expression of antimicrobial peptides (AMPs) at D1 in the lungs and trachea after *S. aureus* infection (**Figures 5A–J**). Indeed, *S. aureus* challenge induced increased expression of Defβ14 (**Figures 5C, D**), RegIIIg (**Figures 5G, H**), and LCN2 (**Figures 5I, J**) in both the lungs and trachea, which have been shown to play important roles in *S. aureus*-induced pneumonia (34–37). Compared with that in the WT infection group, Defβ14 expression in the lungs of mutant mice was significantly lower (**Figure 5C**). In contrast, RegIIIg expression was significantly greater in the lungs of mutant mice than in those of WT-infected mice (**Figure 5G**). Increased expression of cathelicidin (Camp) was observed only in the trachea of WT and mTas2r105^-/-^/104^-/-^/114^-/-^ mice (**Figure 5B**). These results suggest that bitter taste receptor signaling deficiency disrupts the expression dynamics of key AMPs during early S. aureus pneumonia, with tissue- and peptide-specific effects.

Moreover, we investigated the expression profile of mTas2rs in the lungs and trachea after *S. aureus* infection (**Supplementary Figures S3A, B**). In the lungs, pulmonary infection significantly increased the levels of most mTas2rs in mutant and WT mice, similar with previous reports (41). Increased mTas2r135 expression was detected only in WT mice (**Supplementary Figure S3A**). In the trachea, *S. aureus* infection increased the expression of mTas2r106, 126 130, 135, 136 and Gnat3 in WT mice. No significant changes were detected in the mutant mice (**Supplementary Figure S3B**).

### 3.5 Bitter taste signaling deficiency is associated with severe pulmonary lesions during *S. aureus*-induced pneumonia

**Figure 6:**
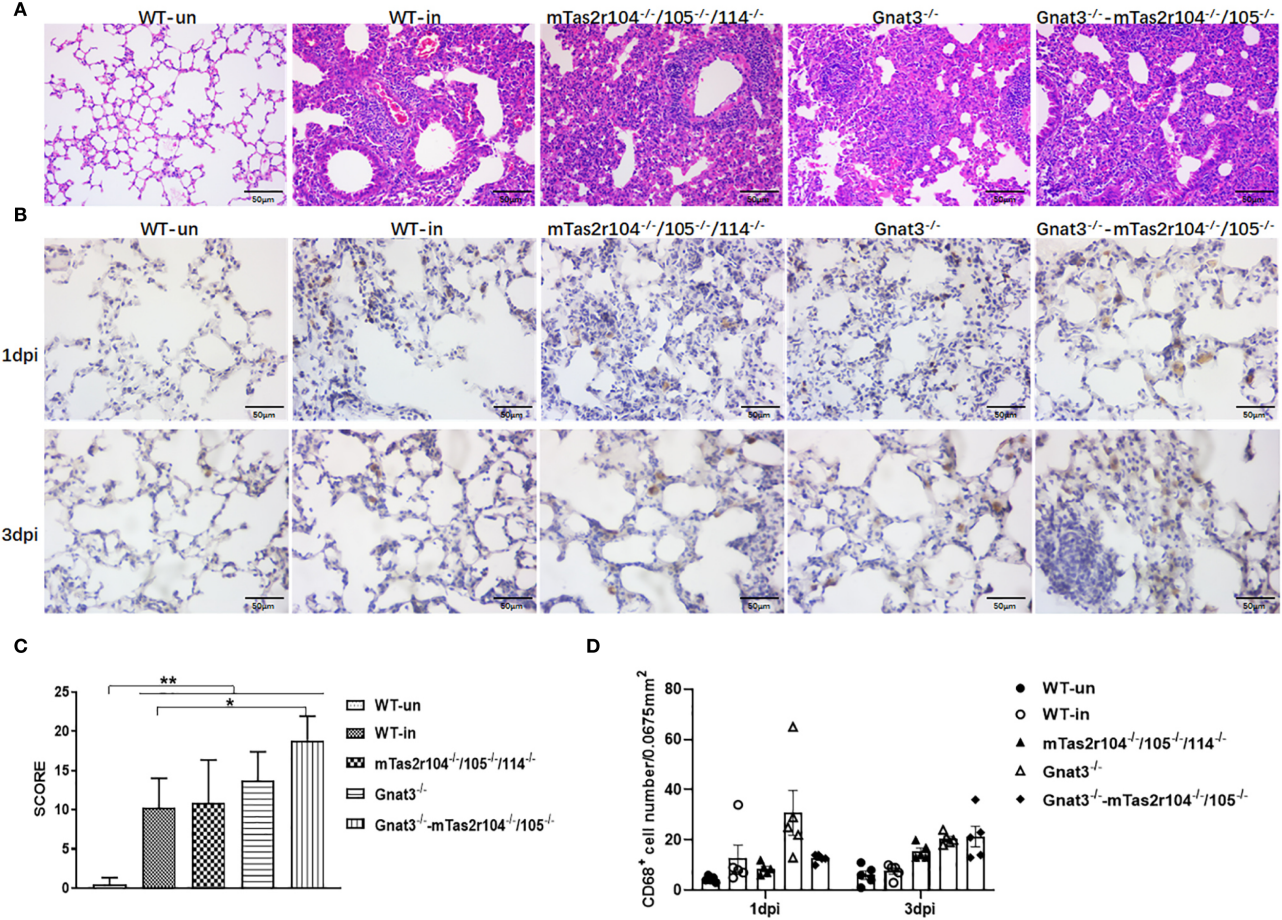
Bitter receptor signaling deficiency leads to increased consolidating pneumonia after the induction of *S. aureus* pneumonia. Representative slides of lung HE-stained sections are shown at D3 post-infection **(A)**. Immunohistochemistry with an anti-CD68 antibody was further performed on 10 μm frozen sections of lung tissues at D1 and D3 post-infection **(B)**. Pathology scores were determined at the indicated time points post-infection in WT mice and mutant mice according to the scoring system described in the Materials and methods. Higher scores were observed in Gnat3-Tas2r104/105 mice **(C)**. The number of CD68^+^ positive cells in each slide was counted. *S. aureus* Newman infection increased the number of CD68^+^ positive cells in D3 lungs **(D)**. The data are shown as the means ± SEMs (n = 3-5). The LSD test was used to analyze significant differences. *P < 0.05, **P < 0.01, ***P < 0.001, ****P < 0.0000. Scale bar 50 μm.

To evaluate the role of the bitter taste signaling pathway in the severity of lung pathology, we analyzed pulmonary inflammation and injury in lung tissue slides at D3 post-infection (**Figures 6A, B**, **Supplementary Figure S4**). Compared with WT mice, Gnat3^-/–^Tas2r104^-/-^/105^-/-^ mice had higher pathology scores (**Figure 6C**). Detailed immunohistochemical analysis with anti-CD68 indicated that CD68+ macrophages had an increasing trend in D3 lung tissues from mutant mice than in those from WT mice (**Figures 6B, D**).

### 3.6 Bitter taste signaling deficiency results in increased Ki67+ cell numbers in the alveolar space and surrounding bronchioles at D3 post-infection

**Figure 7:**
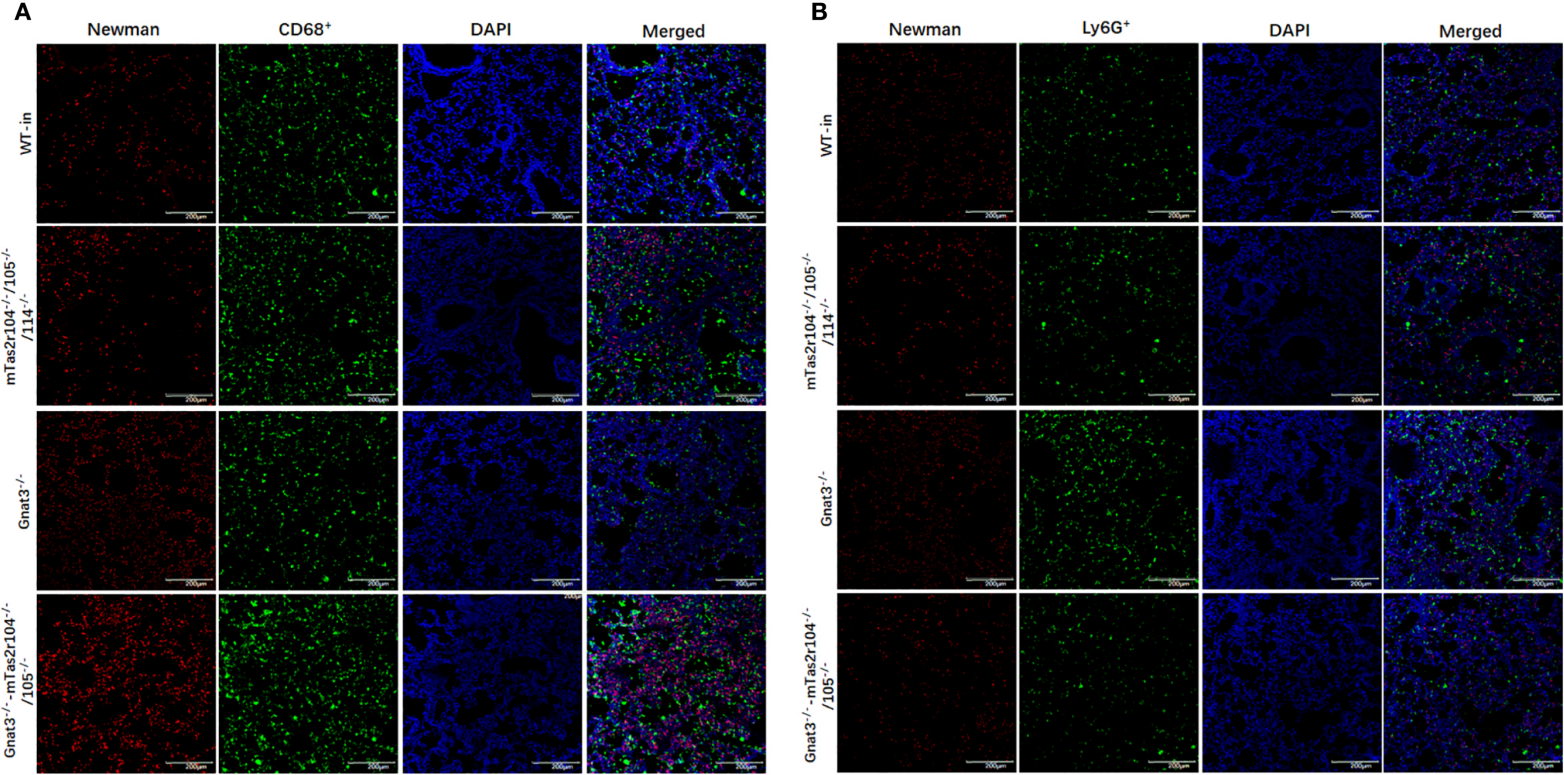
Immunofluorescence staining reveals a large number of *S. aureus* in lung tissues. **(A)** Co-staining with anti-CD68 and anti-S aureus antigens, **(B)** co-staining with anti-Ly6G and anti-S aureus antigens. Scale bar 200 µm.

**Figure 8:**
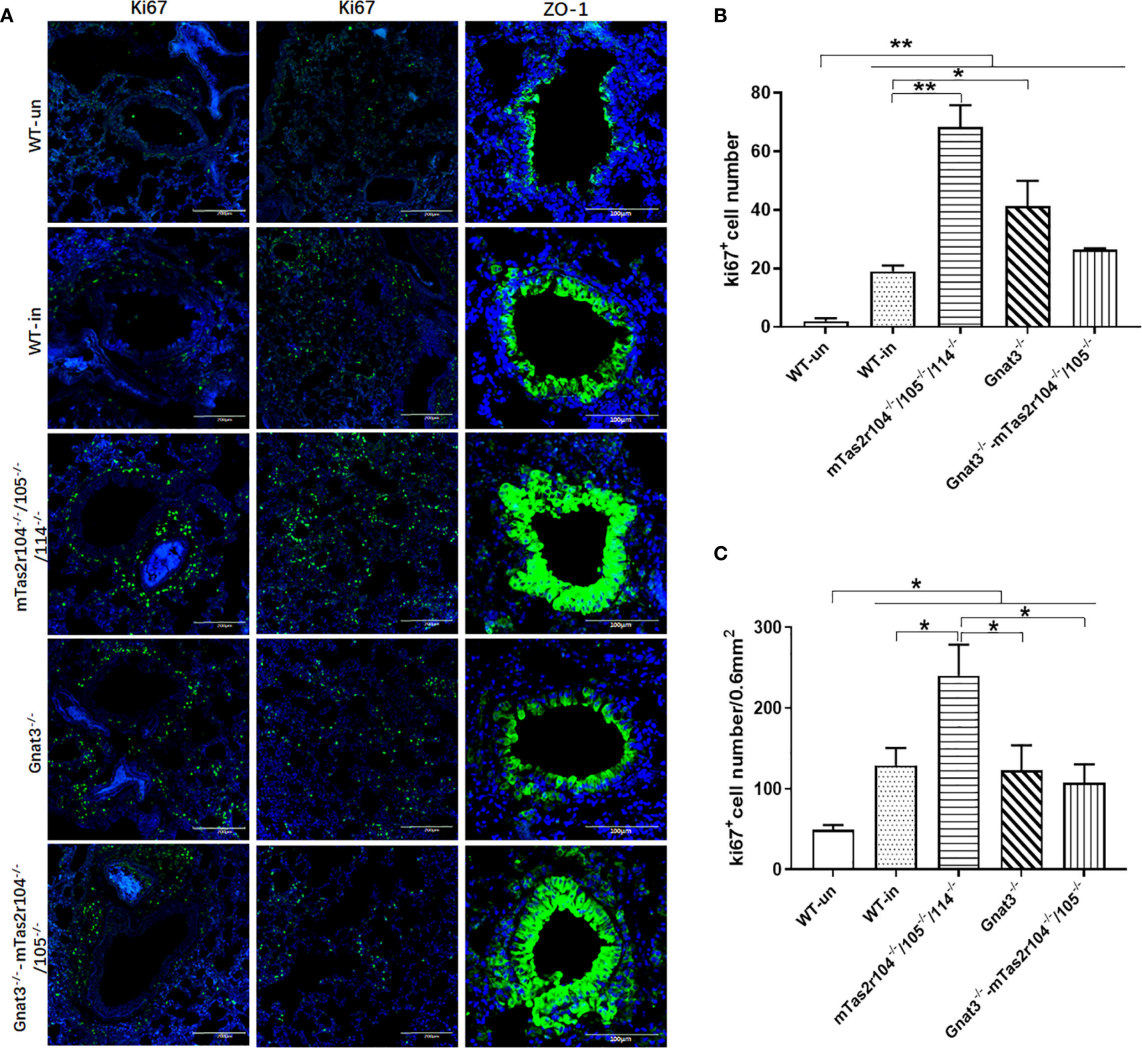
The genetic mutation of bitter taste receptors results in increased Ki67 expression in the alveolar space and surrounding bronchioles at D3 post-infection. Immunofluorescence staining with anti-Ki-67 and anti-ZO-1 antibodies was performed on frozen sections from WT and mutant mice. Representative images are shown. Ki67 expression was significantly upregulated in the surrounding bronchioles (the first column) and alveolar region (the second column) of Tas2r104^-/-^/105^-/-^/114^-/-^ and Gnat3^-/-^ mice. An intense ZO-1-positive signal (the third column) was detected in bronchioles and hypertrophic connective tissue from Tas2r104^-/-^/105^-/-^/114^-/-^ and Gnat3^-/–^mTas2r105^-/-^/114^-/-^ **(A)**. Increased Ki67+ cell numbers in the surrounding bronchioles **(B)** and alveolar space **(C)** were observed. The data are shown as the means ± SEMs. The LSD test was used to analyze significant differences (*P* < 0.05). *P < 0.05, **P < 0.01, ***P < 0.001, ****P < 0.0000. Scale bar 200 µm.

Immunostaining analysis was further employed to investigate the immune response and histological changes in *S. aureus*-induced pneumonia. A large number of *S. aureus* were distributed in lung tissues and very rarely colocalized with CD68+ macrophages (**Figure 7A**) or Ly6G+ neutrophils (**Figure 7B**). Ki-67 is commonly used as a marker to assess cell proliferation (38). Weaker expression of Ki-67 was detected in the lungs of the WT mice (**Figure 8A**). However, Ki-67 expression was significantly upregulated in the surrounding bronchioles (**Figure 8A** the first column and 8B) and alveolar region (**Figure 8A** the second column and 8C) of infected mutant mice, indicating that bitter taste signaling deficiency may affect cell proliferation in the lungs via unknown molecular mechanisms. Ki-67 was colocalized with surfactant protein C (SFTPC), indicating the proliferation of type II alveolar cells (**Supplementary Figure S5**).

Zonalula occludens 1 (ZO-1) expression is clinically associated with inflammation in human lung tissue (39, 40). We also investigated the expression of ZO-1 in infected lung tissues by immunostaining. Representative images are shown in **Figure 8A**. ZO-1 expression was detectable in normal tissues, including bronchioles and alveoli (**Figure 8A** the third column). An intense signal was detected in bronchioles from mTas2r105^-/-^/114^-/-^ and Gnat3-mTas2r104^-/-^/105^-/-^ mice. We also observed a strong signal in hypertrophic connective tissue around bronchioles (**Figure 8A** the third column), indicating that upregulated expression of ZO-1 is associated with increased levels of inflammation in mutant mice.

### 3.7 Bitter taste signaling deficiency activates the mTOR pathway, downregulates eNOS expression and retards the recovery process at D14 post-infection

**Figure 9:**
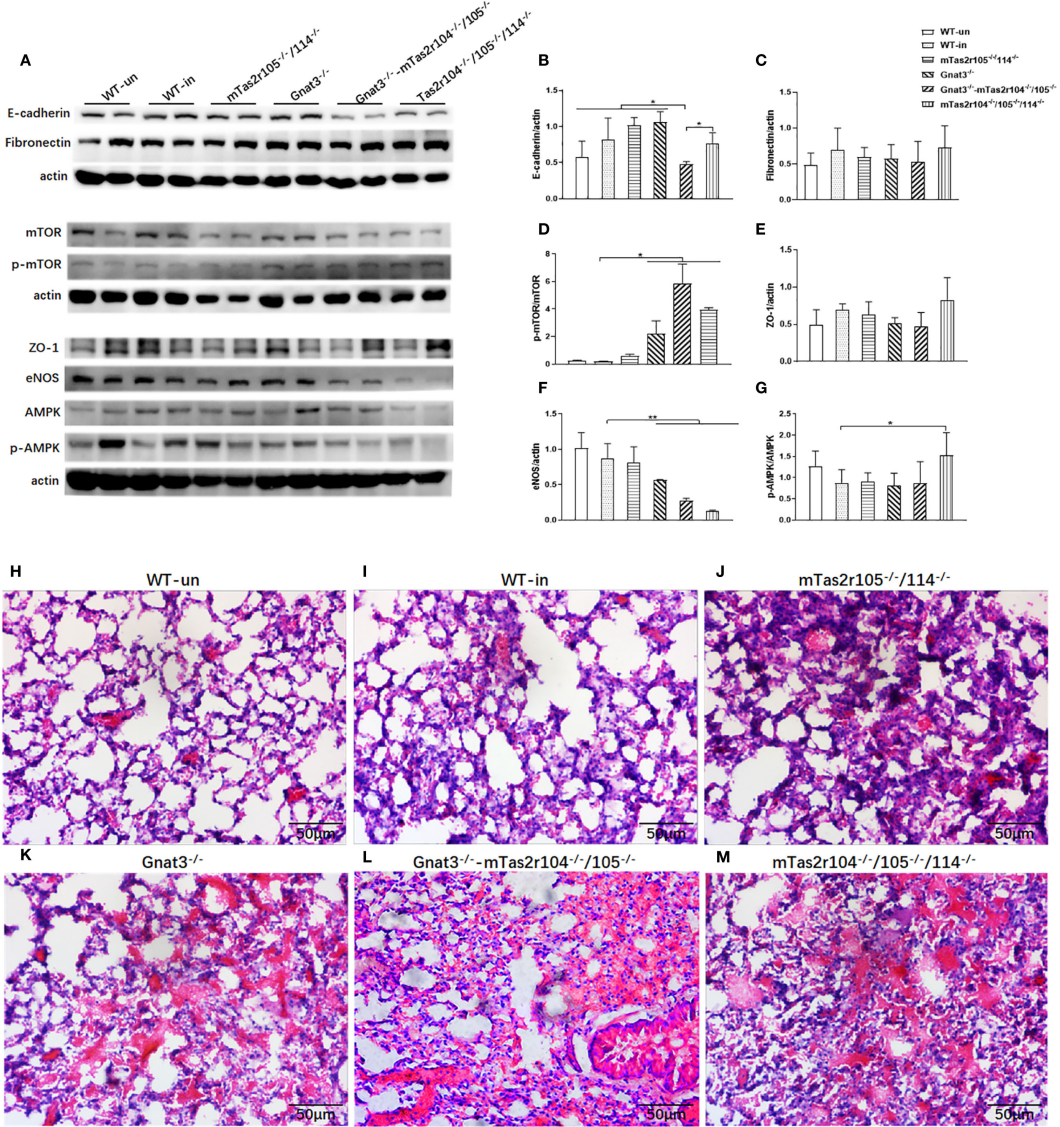
Bitter taste signaling deficiency downregulated the protein expression levels of mTOR and eNOS at D14. Western blotting was used to detect the protein expression levels of E-cadherin, fibronectin, mTOR/p-mTOR, ZO-1, eNOS, and AMPK/pAMPK **(A)**. Band intensities were quantified by spot densitometric analysis via ImageJ software, and the results were normalized to β-actin levels and reported as intensities relative to those of the controls **(B-G)**. E-cadherin **(B)**, fibronectin **(C)**, mTOR/p-mTOR **(D)**, ZO-1 **(E)**, eNOS **(F)**, and AMPK/pAMPK **(G)**. An imbalance in the AMPK **(G)** /mTOR **(D)** pathway and decreased eNOS **(F)** expression were found in mutant mice. The data are shown as the means ± SEMs. The LSD test was used to analyze significant differences (*P* < 0.05). *P < 0.05, **P < 0.01, ***P < 0.001, ****P < 0.0000. Bitter taste signaling deficiency slows the recovery process from pneumonia-induced lung injury. Representative histological images of H&E-stained mouse lungs at D14 post-infection. Severe lung injury was still observed in the mutant mice. **(H)** WT, **(I)** WT mice infected, **(J)** Tas2r105^-/-^/114^-/-^ mice infected, **(K)** Gnat3^-/-^ mice infected, **(L)** Gnat3^-/–^Tas2r105^-/-^/114^-/-^ mice infected, **(M)** Tas2r104^-/-^/105^-/-^/114^-/-^ mice infected. Scale bar 50 µm.

Considering the increased expression of Ki-67 and ZO-1, we speculated that bitter taste signaling deficiency could influence the expression of several critical pathways in lung tissues from infected mice at D14 post-infection (**Figures 9A–G**). No significant changes in E-cadherin (**Figure 9B**), fibronectin (**Figure 9C**) or ZO-1 (**Figure 9E**) were detected via western blot analysis. However, the expression level of the mTOR protein was significantly decreased, and the phosphorylation level of the mTOR protein was significantly increased in the mutant mice compared with the WT-infected mice (**Figure 9D**). Interestingly, we also observed a significant decrease in the level of the endothelial nitric oxide synthase (eNOS) protein in Gnat3^-/–^mTas2r104^-/-^/105^-/-^ and mTas2r104^-/-^/105^-/-^/114^-/-^ mice compared with WT mice (**Figure 9F**). The ratio of p-AMPK/AMPK was significantly increased in mTas2r104^-/-^/105^-/-^/114^-/-^ mice compared with WT-infected mice (**Figure 9G**). Representative histological images of H&E-stained mouse lungs revealed thickened alveolar walls and hemorrhagic lesions in mutant mice (**Figures 9H–M**).

**Figure 10:**
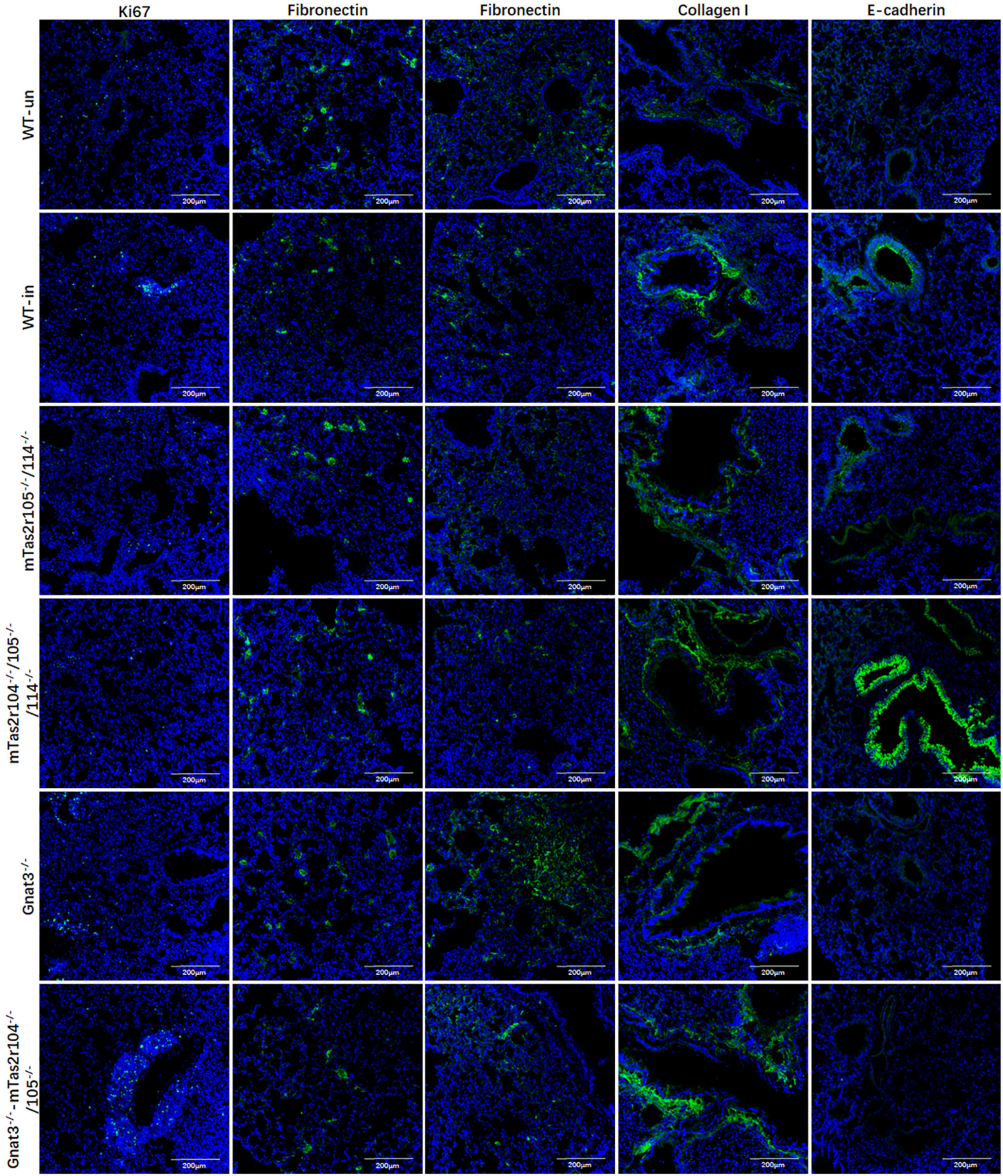
Immunofluorescence staining for Ki67, fibronectin, collagen I, and E-cadherin in lung tissues at D14 post-infection. Antibody and mice lines are shown in image. First column for ki67, second for fibronectin in alveolar region, third for fibronectin in bronchial region, fourth for collagen I in bronchial region, fifth for E-cadherin in bronchial region. Noted that intense signals in connective tissue for collagen I for mutant mice, and intense signals for E-cadherin were observed in bronchioles from Tas2r104^-/-^/105^-/-^/114^-/-^ mice. Scale bar 200 µm.

In addition, immunofluorescence staining for Ki67, fibronectin, collagen I and E-cadherin was further performed in lung tissues at D14 post-infection (**Figure 10**).

Ki67 was less expressed in alveolar region in all groups (**Figure 10** the first column). We can observe the increased expression of Ki67 in proliferation tissues (**Figure 10** the first column). The histological distributions of fibronectin in alveolar region (**Figure 10** the second column) or bronchial region (**Figure 10** the third column) were not significantly different between the mutant and WT mice. The expression of collagen I increased in connective tissues of bronchiole from these mutant mice (**Figure 10** the fourth column). We observed intense signals for E-cadherin in bronchioles from Tas2r104^-/-^/105^-/-^/114^-/-^ mice (**Figure 10** the fifth column). Collectively, these findings demonstrate that bitter taste signaling deficiency leads to the activation of mTOR pathway, reduces eNOS expression, and retards the recovery process at D14 post-infection.

## 4 Discussion

Although bitter taste receptors are considered to be expressed in the airways of both mice and humans, the characteristics of bitter taste receptors are still controversial. With a Tas2r143-CreERT2 transgenic mouse line, Tas2r105/106 transcripts were found in Tas2r143-positive cells from the epithelium of the trachea and primary bronchi (31). However, the expression of the Tas2r105 gene cluster was not detected in the airway in another study (30). H1N1 infection regulate the expression level of Tas2rs including Tas2r105 and Tas2r143 in lung tissue, Gγ13-mediated bitter signals play a critical role in tuft cells-mediated inflammation resolution and functional repair of the damaged lungs (41). In this study, the expression of the Tas2r105 gene was observed in the trachea and primary bronchi. Interestingly, infection increased the expression levels of the Tas2r105 and Tas2r143 genes in the lungs of WT and mutant mice. The differential expression of the two gene clusters in the trachea and lung may be due to tissue-specific transcriptional regulation post-infection. A previous study revealed increased expression of bitter receptors in respiratory epithelial cells post-infection (16, 23). In the respiratory system, activation of bitter taste receptors has been suggested to be related to innate immunity (16, 22). Consistent with previous finding that disruption of the bitter taste signaling pathway delays the resolution of pneumonia-induced lung injury (41), these two gene clusters may be functionally related to the innate immune response in the lung.

Here, another finding was the decreased expression of IL-10 and MIP-2 in bitter receptor signaling-deficient mice. Interleukin-10 (IL-10) is an important anti-inflammatory cytokine produced under different conditions of immune activation by a variety of cell types, including T cells, B cells, and monocytes/macrophages.

Macrophage inflammatory protein (MIP)-2 plays a significant role in respiratory tract defenses and contributes to the pathogenesis of inflammatory lung disease by recruiting neutrophils to the site of infection. A recent study revealed that bitter taste receptor signals are involved in the inhibition of the release of LPS-induced cytokines, including TNF-α, CCL3, CXCL8, and IL-10 in human lung macrophages (18). TAS2Rs also significantly inhibit the release of histamine and PGD2 from IgE receptor-activated primary human mast cells (20). Activation of TAS2R16 signaling by salicin inhibited the release of lipopolysaccharide (LPS)-induced proinflammatory cytokines, at least in part, by repressing LPS-induced intracellular cAMP elevation and NF-kB p65 nuclear translocation in human gingival fibroblasts (42).

In addition, bitter taste signaling deficiency inhibited cathelicidin expression in the airway and decreased the expression of Defβ14, Hamp and LCN2 in the lungs. The level of upregulated genes also varied among the WT and mutant mice. Elevated RegIIIg in mutants could reflect an attempt to counter balance Defβ14 deficiency, as RegIIIg targets Gram-positive bacteria. These findings collectively indicate that bitter taste receptor signaling deficiency disrupts the expression dynamics of key antimicrobial peptides (AMPs) during early S. aureus pneumonia, with tissue specific effects. AMPs are important effectors of innate immunity because of their immunomodulatory activity and direct killing of microorganisms at mucosal surfaces and are involved in the immunopathogenesis of several infectious diseases (37). AMPs are upregulated in airways during bacterial infection and have been detected in airway surface fluid, bronchoalveolar lavage fluid (BALF), alveolar macrophages, neutrophils, and airway epithelial cells (35, 36). During the early stages of infection, cathelicidin is synthesized by resident alveolar macrophages and airway epithelial cells; neutrophils are subsequently recruited and produce cathelicidin and a-defensins (43). Given that taste receptor signaling plays an important role in regulating the function of airway epithelial cells, alveolar macrophages and neutrophils (17, 19, 23), the findings of the present study collectively suggest that taste receptor signaling may be involved in the regulation of AMPs and cytokine expression in the respiratory tract and lung by affecting the biological function of these cells. The molecular mechanisms by which bitter taste receptor signaling regulates AMPs and cytokine expression in the lungs should be further investigated.

The importance of bitter taste receptor signaling in repair after acute lung injury is supported by a further study showing a decreased expression level of eNOS, increased level of p-mTOR, and severe pathological damage in mutant mice. Previous studies have revealed that the eNOS-NO pathway plays a pivotal role in fetal lung vascular development and lung morphogenesis (44). In mouse models of bronchopulmonary dysplasia (BPD), the expression of the protein lung endothelial nitric oxide synthase (eNOS) is reduced, suggesting that eNOS is important in the regulation of lung alveolar and vascular growth (44). Inhaled nitric oxide (NO) may protect against changes in lung structure in rat models of BPD (45). eNOS directly participates in lung fibrosis resolution. Loss of eNOS in human lung ECs reduced the suppression of TGFβ-induced lung fibroblast activation in 2D and 3D cocultures (46). Previous study have shown that mTOR played a crucial role in regulating *S. aureus* infection and the levels of chemokines (47). Activation of mTOR also contributed to proliferative lung diseases such as lung cancer and pulmonary hypertension (48, 49). Another study further indicated that mTOR activation could drive cell senescence, generate the senescence-associated secretory phenotype (SASP), and induce emphysema and pulmonary hypertension (50). mTOR activation in microvascular progenitor cells (MVPC) disrupt lung microvascular endothelial barrier function and tissue structure, and multiple developmental signaling pathways (51).

TAS2Rs belong to the family of G protein-coupled receptors (GPCRs). They not only participate in taste perception but also regulate cell functions in tissues such as the respiratory tract via downstream signaling molecules (e.g., cyclic adenosine monophosphate (cAMP), calcium ions (Ca^2+^), Protein Kinase A (PKA), etc.) (52–54).

TAS2Rs may indirectly inhibit mTOR activation by suppressing the Phosphoinositide 3-Kinase (PI3K)/Akt pathway (55–57). After bitter taste signaling knockout, the attenuation of the inhibitory regulation may result in increased mTOR phosphorylation levels.

Bitter taste receptors may directly or indirectly promote the expression/activity of endothelial Nitric Oxide Synthase (eNOS) (17, 23, 58). For instance, bitter taste signals can enhance the phosphorylation (activation) or transcription of eNOS (e.g., via transcription factors such as cAMP Response Element-Binding Protein (CREB)) by increasing intracellular Ca^2+^ levels or activating Protein Kinase A (PKA). Thus, bitter taste signaling deficiency can result in decreased eNOS expression (23, 58). In addition, eNOS/NO pathway may inhibit the kinase activity of mammalian Target of Rapamycin (mTOR) by a negative feedback regulation (59). The inhibition of eNOS/NO pathway in mutant mice relieves the negative feedback regulation, ultimately causing continuous mTOR activation.

In short, at the late stage of Staphylococcus aureus (S. aureus) infection (Day 14), the mTOR activation and eNOS reduction induced by bitter taste receptor (TAS2R) knockout is the product of the interactions among many factors including the infected microenvironment, crosstalk between signaling pathways, and loss of TAS2R-mediated regulation. This inverse change may reflect an adaptive response of the host: in mutant mice, the host enhances mTOR-mediated repair/immune functions to compensate for the defects of the eNOS/NO pathway (e.g., insufficient antibacterial activity, impaired anti-inflammatory effects, and compromised vascular regulation). However, it may also exacerbate local tissue damage (e.g., vascular dysfunction, oxidative stress). The phenomenon discovered in this study (delayed repair of lung injury, disfunction of mTOR pathway and eNOS/NO pathway in mutant mice) remain to be clarified. Further studies are therefore warranted to explore the potential relationship between the mTOR pathway, the eNOS/NO signal, and bitter taste receptor signal in the process of repair of lung injury. Further verification requires combining cell-specific analyses (e.g., endothelial cells vs. immune cells) and signal pathway inhibition experiments (e.g., observing eNOS changes after mTOR inhibition).

In addition, the different expression patterns of molecular markers in lung tissues from mTas2r105^-/-^/114^-/-^ and mTas2r104^-/-^/105^-/-^/114^-/-^ mice also indicate that these three receptors may perform different biological functions during repair after lung injury. Many factors, such as epithelial cells (60, 61), endothelial cells (62), neutrophils (63) and macrophages (64, 65,) are involved in the process of repair after lung injury. The results of the present study revealed the expression of bitter taste receptor signaling in these cells (58). Key taste transduction genes, including bitter taste receptors, the G protein gustducin and the gustatory ion channel TRPM5 (M5), are found in the ventricular walls of the murine brain and play critical roles in regulating glucose homeostasis (66). Another study revealed TAS2R expression in the hematopoietic stem/progenitor cell (HSPC) compartment and that activation of TAS2Rs by denatonium benzoate in various cell types can modulate hematopoietic stem cell fate (67). T2R10, T2R14 and T2R38 are highly expressed in human pulmonary arterial ECs. Bitter taste sensing in the pulmonary endothelium to regulate barrier integrity *in vitro* through cAMP-Rac1 signaling. The bitter-taste agonists’ phenylthiourea and denatonium protect the pulmonary endothelium against LPS-induced barrier disruption through T2R38 and T2R10, respectively (58). Bitter receptor signaling deficiency may affect the physiological function of these cells (epithelia, endothelial and immune cells), ultimately contributing to repair after lung injury in a receptor expression-dependent manner.

In short, TAS2Rs emerge as multifaceted modulators of airway immunity, balancing antimicrobial defense, inflammation, and tissue repair. While not essential for survival, their signaling fine-tunes responses to infection, highlighting potential therapeutic targets for respiratory infections.

## Data Availability

The datasets presented in this study can be found in online repositories. The names of the repository/repositories and accession number(s) can be found in the article/**Supplementary Material**.

## Supplementary material

Supplementary Figure 1 Bitter taste signaling deficiency leads to more severe weight loss after *S. aureus*-induced pneumonia. WT and mutant mice were intranasally inoculated with 10^9^ bacteria or received an equivalent volume of PBS. **(A)** All mice, including Tas2r104^-/-^/105^-/-^, Tas2r105^-/-^/114^-/-^, Tas2r104^-/-^/105^-/-^/114^-/-^ and WT mice, were weighed daily for 7 days to monitor weight loss (n = 20-25). One hundred percent corresponds to the initial weight. Infected animals were compared with WT mice receiving PBS. **(B)** Survival curves during infection with the Newman strain (n = 20-25). **(C)** All Tas2r105^-/-^/114^-/-^, Tas2r104^-/-^/105^-/-^/114^-/-^, Gnat3^-/-^, Gnat3^-/–^Tas2r104^-/-^/105^-/-^ and WT mice were weighed daily for 14 days to monitor weight loss (n = 12-15). One hundred percent corresponds to the initial weight. Infected animals were compared with WT mice receiving PBS.

Supplementary Figure 2 qRT-PCR analysis of the mRNA expression of cytokines and *S. aureus* virulence factors in the lungs after *S. aureus* Newman infection. The protein expression of INF-γ **(A)**, TNFα **(B)** and IL-6 **(C)** in lung homogenates at D1 and D3 post-infection. The expression of 16S **(C)**, SEA **(D)** and RNAIII **(E)** in lung homogenates at D1 and D3 post-infection. **(F)** Bacterial load in lung homogenates. The data are shown as the means ± SEMs (n = 4-5). The LSD test was used to analyze significant differences (*P* < 0.05). **P* < 0.05, ***P* < 0.01, ****P* < 0.001, *****P* < 0.0000.

Supplementary Figure 3 Expression levels of bitter taste receptors after *S. aureus* Newman infection. The expression profile of bitter taste receptors in the lungs **(A)** and trachea **(B)**. The data are shown as the means ± SEMs (n = 4-5). The Dunnett test, or least significant difference (LSD) test, was used to analyze significant differences (*P* < 0.05). **P* < 0.05, ***P* < 0.01, ****P* < 0.001, *****P* < 0.0000.

Supplementary Figure 4 Representative histology of whole-lung sections from WT and mutant mice at D3 post-infection. The mouse strain is labeled in the figure, with the left image showing a panoramic view and the right image showing an enlarged view.

Supplementary Figure 5 Representative images of immunofluorescence staining of SFTPC and Ki67. Ki67 (green), SFTPC (red), DAPI (blue) and colocalization in the lung, scale bar = 200 µm.
