# Supplementary figures and images for: Contribution of the bitter taste signaling pathway to lung inflammation during Staphylococcus aureus-induced pneumonia
Source: Front Immunol. 2025 Oct 9;16:1647780. doi: 10.3389/fimmu.2025.1647780 (PMC12545109; doi:10.3389/fimmu.2025.1647780)

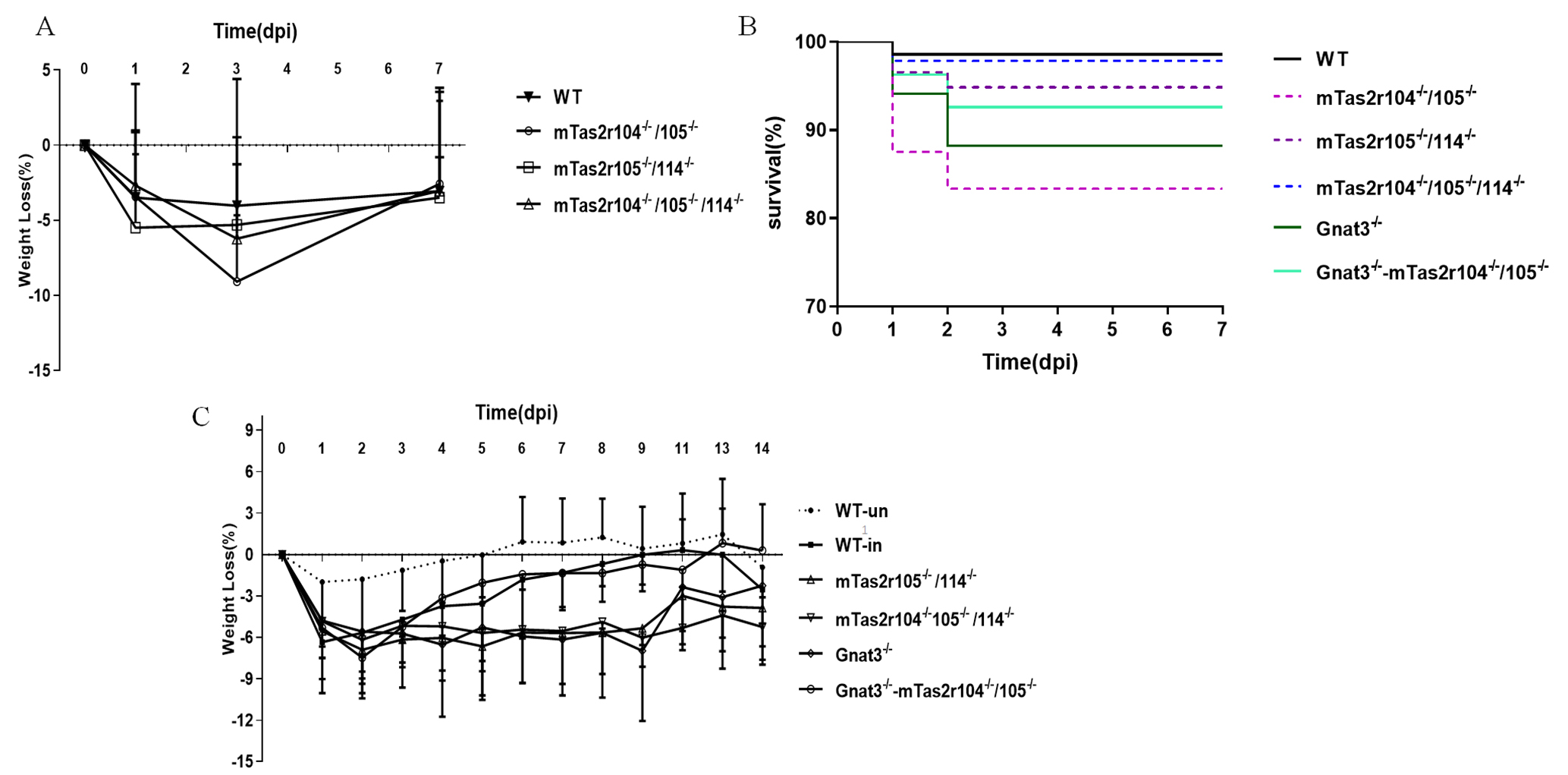

Supplement: Supplementary Figure 1 — Bitter taste signaling deficiency leads to more severe weight loss after S. aureus-induced pneumonia. WT and mutant mice were intranasally inoculated with 109 bacteria or received an equivalent volume of PBS. (A) All mice, including Tas2r104-/-/105-/-, Tas2r105-/-/114-/-, Tas2r104-/-/105-/-/114-/- and WT mice, were weighed daily for 7 days to monitor weight loss (n = 20-25). One hundred percent corresponds to the initial weight. Infected animals were compared with WT mice receiving PBS. (B) Survival curves during infection with the Newman strain (n = 20-25). (C) All Tas2r105-/-/114-/-, Tas2r104-/-/105-/-/114-/-, Gnat3-/-, Gnat3-/–Tas2r104-/-/105-/- and WT mice were weighed daily for 14 days to monitor weight loss (n = 12-15). One hundred percent corresponds to the initial weight. Infected animals were compared with WT mice receiving PBS. [file Image1.jpeg]

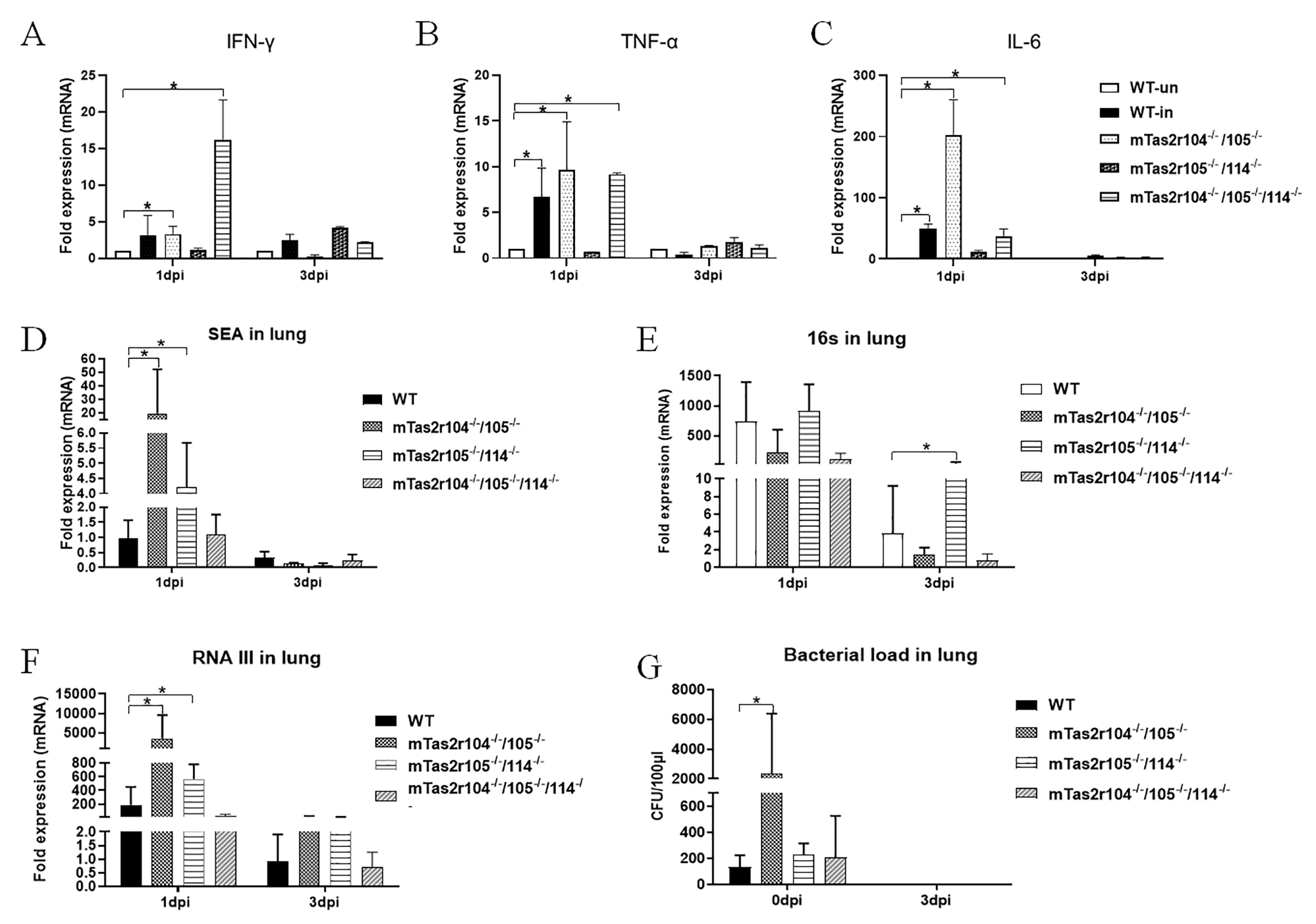

Supplement: Supplementary Figure 2 — qRT-PCR analysis of the mRNA expression of cytokines and S. aureus virulence factors in the lungs after S. aureus Newman infection. The protein expression of INF-γ (A), TNFα (B) and IL-6 (C) in lung homogenates at D1 and D3 post-infection. The expression of 16S (C), SEA (D) and RNAIII (E) in lung homogenates at D1 and D3 post-infection. (F) Bacterial load in lung homogenates. The data are shown as the means ± SEMs (n = 4-5). The LSD test was used to analyze significant differences (P < 0.05). *P < 0.05, **P < 0.01, ***P < 0.001, ****P < 0.0000. [file Image2.jpeg]

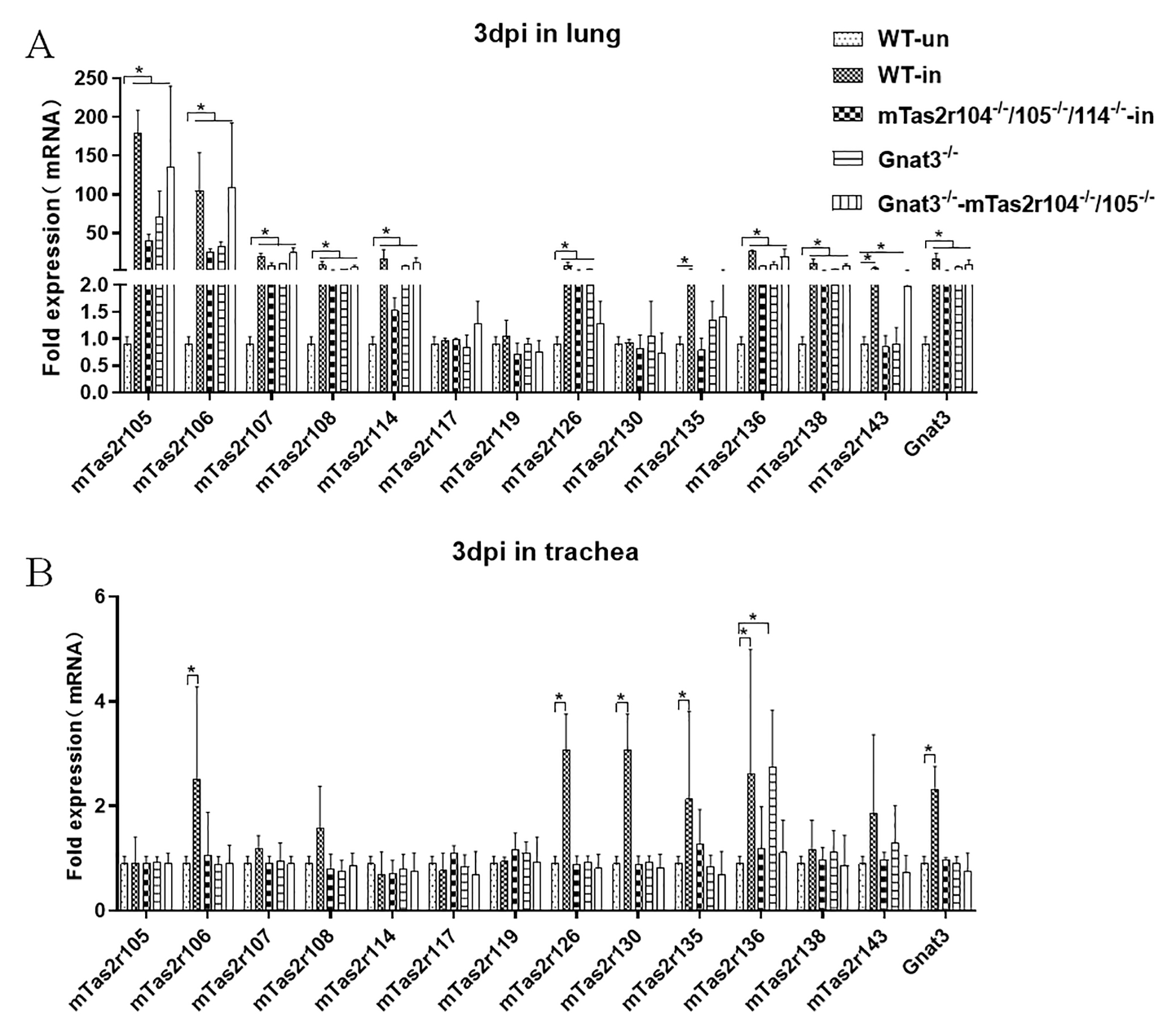

Supplement: Supplementary Figure 3 — Expression levels of bitter taste receptors after S. aureus Newman infection. The expression profile of bitter taste receptors in the lungs (A) and trachea (B). The data are shown as the means ± SEMs (n = 4-5). The Dunnett test, or least significant difference (LSD) test, was used to analyze significant differences (P < 0.05). *P < 0.05, **P < 0.01, ***P < 0.001, ****P < 0.0000. [file Image3.jpeg]

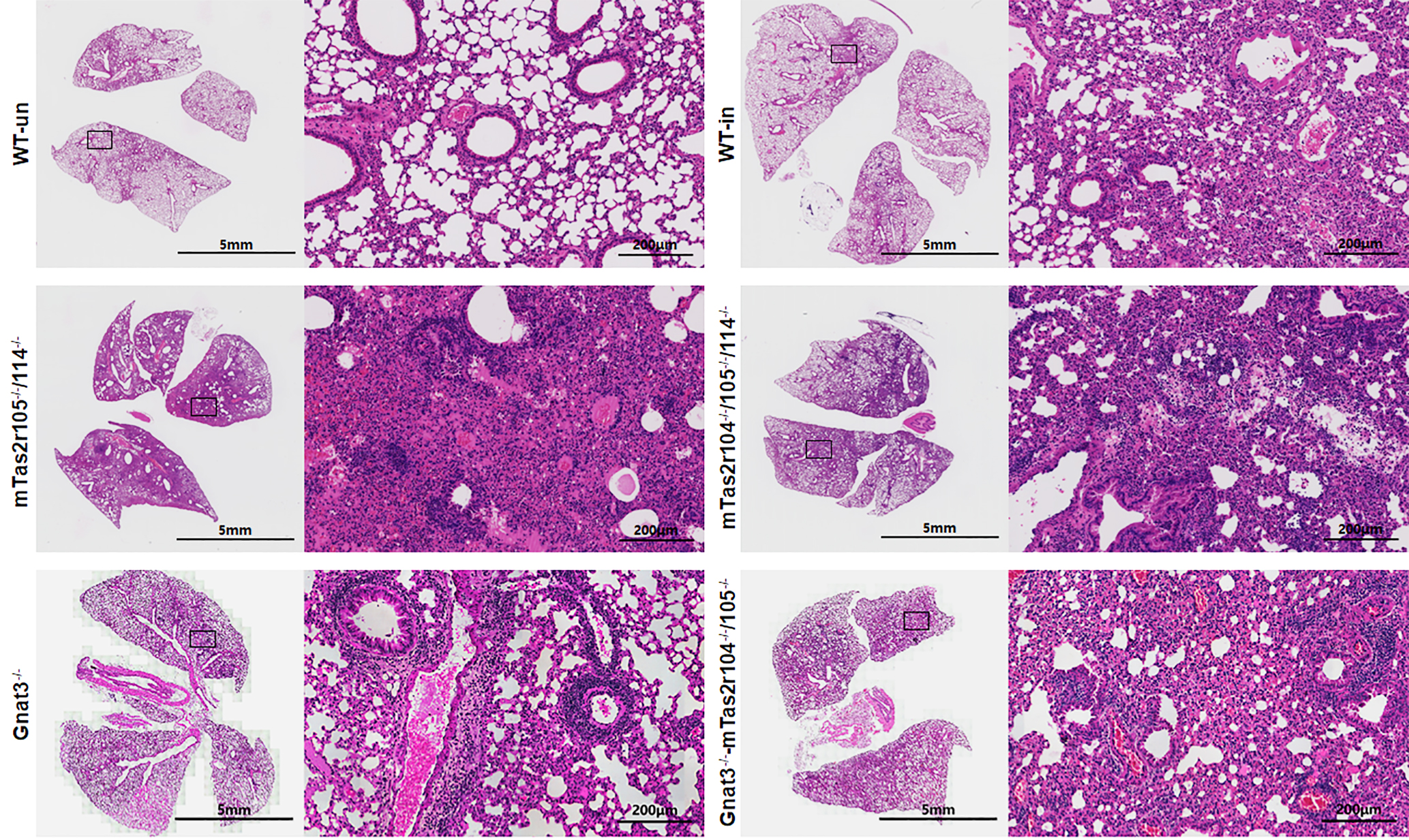

Supplement: Supplementary Figure 4 — Representative histology of whole-lung sections from WT and mutant mice at D3 post-infection. The mouse strain is labeled in the figure, with the left image showing a panoramic view and the right image showing an enlarged view. [file Image4.jpeg]

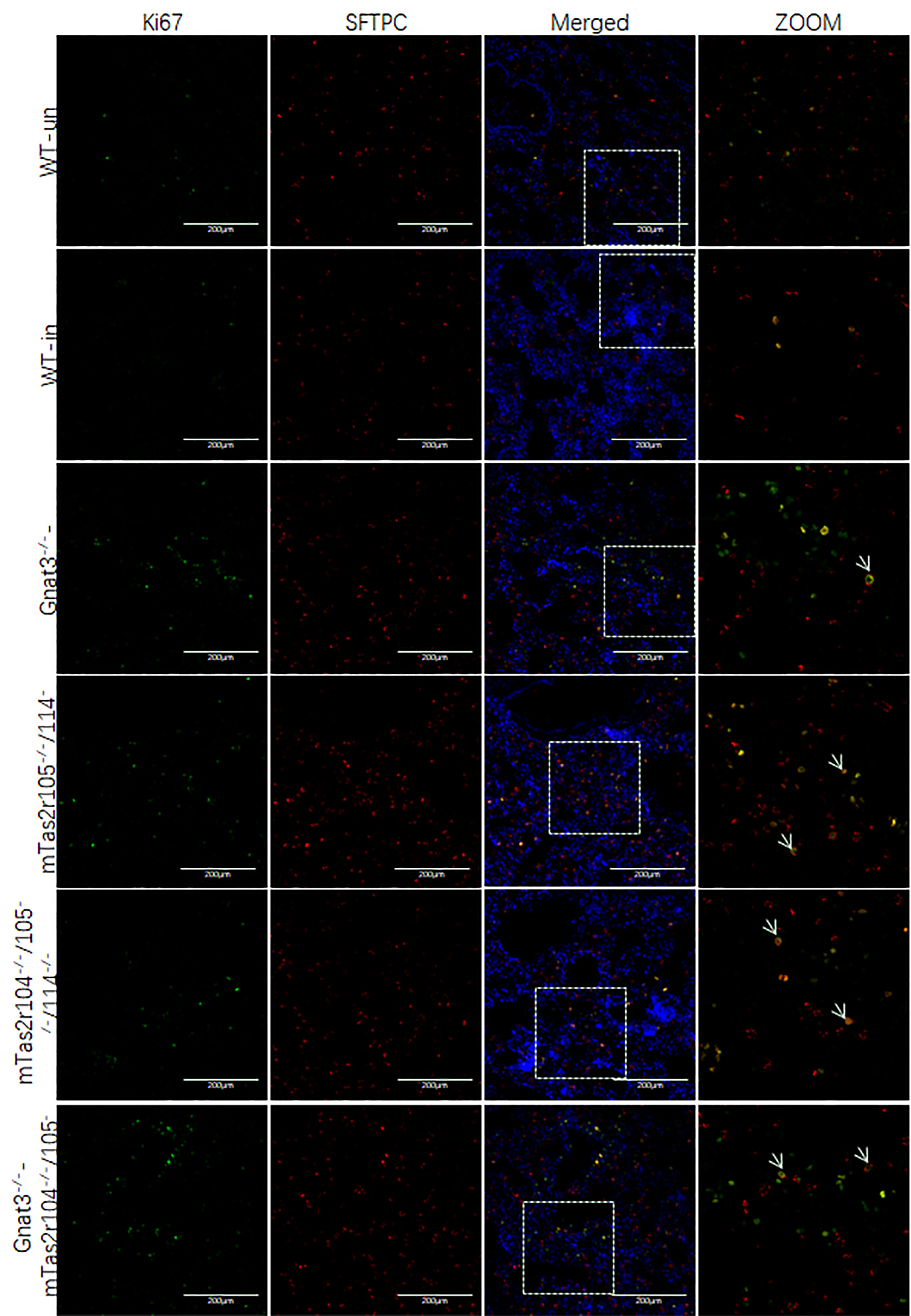

Supplement: Supplementary Figure 5 — Representative images of immunofluorescence staining of SFTPC and Ki67. Ki67 (green), SFTPC (red), DAPI (blue) and colocalization in the lung, scale bar = 200 µm. [file Image5.jpeg]
