# Supplementary material for: Contribution of the bitter taste signaling pathway to lung inflammation during Staphylococcus aureus-induced pneumonia
Source: Front Immunol. 2025 Oct 9;16:1647780. doi: 10.3389/fimmu.2025.1647780 (PMC12545109; doi:10.3389/fimmu.2025.1647780)
Supplement: Supplementary file 6 [file Table1.docx]

| Oligonucleotides used for real-time quantitative PCR of detecting mTas2r gene. | | |
| --- | --- | --- |
| Gene | Forward Oligonucleotide (5’-3’) | Reverse Oligonucleotide(5’-3’) |
| mTas2r-105-QPCR | CAGAGACCTCAACACAGAA | ATGAGTGACAGCAAGGATAT |
| mTas2r-106-QPCR | CTCACAGGCTTGGCTATT | CAGGAGATAGAAGAGGTTGA |
| mTas2r-107-QPCR | TCCCTGCGGTCACTCAATCATC | CAGTGCCTTCAAAGAGGCTTGC |
| mTas2r-108-QPCR | ACAGTCGCAGAATTGCCTCTCC | AGGAATCTAGTGATGGCCAAGCTG |
| mTas2r-114-QPCR | GCTGTCTCCTGTCAACATAA | AACCATCTTCGCAACAACT |
| mTas2r-117-QPCR | CACTGTTGGTGTCATTGCTCC | GGACAAAACAACGGGGACAG |
| mTas2r-119-QPCR | TCACACCCACAAGAAGGAGCAC | ACCTTAAGGATGGAGAACCTGCAC |
| mTas2r-126-QPCR | CCCGGCAGCTCATTAGTCTT | CGGACACCAAGATAGAGCCC |
| mTas2r-130-QPCR | GACAGAGGCATGTCCAGCTT | CCACCTGCCTCAGCATTTTC |
| mTas2r-135-QPCR | TCAGTTCTGCCAGCAACACACC | TGAATCACCACCTGCCACATCC |
| mTas2r-136-QPCR | CACTCTAGGCAGTTTAGTTCCCT | AGCCTCATTGTCCTAAGGTGTT |
| mTas2r-137-QPCR | AGCATACATTTGTGGCCATGCTC | AAGCAGAGGGTCCCTTAGATCCAG |
| mTas2r-138-QPCR | TGCTATTCAGCTCGCCTGCTTC | TGGCTTGGTAGTTGTGGCTCAG |
| mTas2r-140-QPCR | CATGCAACACAATGCCAAAGACTC | AGGGCCTTAATATGGGCTGTGG |
| Gnat3-QPCR | TAGGAGCCGAGAGGACCAAG | GCTGGTATTCAGATGCCCTTTC |
| Oligonucleotides used for real-time quantitative PCR of detecting cytokines and other genes. | | |
| Gene | Forward Oligonucleotide (5’-3’) | Reverse Oligonucleotide(5’-3’) |
| IL-4-QPCR | TCACAGCAACGAAGAACA | GTGGACTTGGACTCATTCA |
| IL-6-QPCR | CCGCTATGAAGTTCCTCTC | GGTATCCTCTGTGAAGTCTC |
| IL-10-QPCR | GAGCAGGTGAAGAGTGATT | TCCAGCAGACTCAATACAC |
| IL-12p40-QPCR | CAGAAAGGTGCGTTCCTCGTA | AAGCCAACCAAGCAGAAGACAG |
| IFN-γ-QPCR | AGCAACAGCAAGGCGAAAA | CTGGACCTGTGGGTTGTTGA |
| TNF-α-QPCR | CTTCTCATTCCTGCTTGTGG | ATCTGAGTGTGAGGGTCTGG |
| MCP-1-QPCR | CTCTTCCTCCACCACCAT | CTCTCCAGCCTACTCATTG |
| MIP-2-QPCR | GCAAGGCTAACTGACCTGGAA | CAACATCTGGGCAATGGAAT |
| Camp-QPCR | ATCACTGCTGCTGCTACT | CTCTGCCTTGCCACATAC |
| Hamp-QPCR | CCAGCAACAGATGAGACA | ACAACAGATACCACAGGAG |
| Defβ14-QPCR | CATTCCTACCAAAAACCCTC | CTTCTACTTCTTCTTTCGGC |
| RegIIIg-QOCR | CCGTATAACCATCACCATCA | AACAAGGCATAGCAATAGGA |
| Lcn2-QPCR | ATATGCACAGGTATCCTCAG | GAAACGTTCCTTCAGTTCAG |
| Sea-QPCR | TTGGAAACGGTTAAAACGAA | GAACCTTCCCATCAAAAACA |
| 16s-QPCR | GTAGGTGGCAAGCGTTAT | CATCAGCGTCAGTTACAGA |
| RANIII-QPCR | CCATTTTACTAAGTCACTGATTGTT | TGATGGAAAATAGTTGATGAGTTGT |
| mβ2m-QPCR | GAGGCTATCCAGCGTACTCCA | CGGCAGGCATACTCATCTTTT |
| H2-AB1-QPCR | CCGTCACAGGAGTCAGAA | GAGGTGGTGGATACAATAGTA |
| H2-EB1-QPCR | CAAGATGTTGAGTGGAGTTG | GAGTCCTGTTGGCTGAAG |
