# Supplementary material for: Contribution of the bitter taste signaling pathway to lung inflammation during Staphylococcus aureus-induced pneumonia
Source: Front Immunol. 2025 Oct 9;16:1647780. doi: 10.3389/fimmu.2025.1647780 (PMC12545109; doi:10.3389/fimmu.2025.1647780)
Supplement: Supplementary file 7 [file Table2.docx]

| **protein name** | **antibody** | **Company** | **Item No** | **ratio in WB** | **ratio in IHC** |
| --- | --- | --- | --- | --- | --- |
| Newman | Rabbit Anti-Staphyloccocus aureus Rosenbach  tropina/RBITC Conjugated antibody | bioss | bs-4582R-RBITC |  | 1：600 |
| CD68 | Anti -CD68 Rabbit pAb | Servicebio | GB11067 |  | 1：800 |
| Ly6g | Anti -Ly6g Rabbit pAb | Servicebio | GB11229 |  | 1：800 |
| Collagen I | Anti - Collagen I Rabbit pAb | Servicebio | GB11022-3 |  | 1：800 |
| ki67 | Anti -Ki67 Rabbit pAb | Servicebio | GB111141 |  | 1：800 |
| SFTPC | Anti - SFTPC Polyclonal antibody | Proteintech | 10774-1-AP |  | 1：800 |
| ZO-1 | Anti - ZO1 tight junction protein Rabbit pAb | Servicebio | GB111402 | 1：1000 | 1：800 |
| E cadherin | Anti - E Cadherin Rabbit pAb | Servicebio | GB11082 | 1：1000 | 1：800 |
| Fibronectin | Anti- Fibronectin Rabbit pAb | Servicebio | GB114491 | 1：1000 | 1：800 |
| mTOR | Anti -mTOR Rabbit pAb | Servicebio | GB111839 | 1：1000 |  |
| p-mTOR | Anti- Phospho-mTOR (S2481) Rabbit pAb | Servicebio | GB114489 | 1：1000 |  |
| eNOS | Anti-eNOS Rabbit pAb | Servicebio | GB11086 | 1：500 |  |
| AMPK | Anti -AMPK alpha 1 Rabbit pAb GB112669 | Servicebio | GB112669 | 1：1000 |  |
| p-AMPK | Anti- Phospho-AMPK alpha 1 (T183) +  AMPK alpha 2 (T172) Rabbit pAb | Servicebio | GB114323 | 1：1000 |  |
| β-Actin | Recombinant Anti - beta Actin antibody ( Rabbit mAb ) | Servicebio | GB15003 | 1：1500 |  |
|  | ProteinFind® Goat Anti-Rabbit IgG (H+L), HRP Conjugate | Transgene | HS101-01 | 1：5000 |  |
|  | Goat anti-Rabbit IgG (H+L) Cross-Adsorbed Secondary  Antibody, Alexa FluorTM488 | Invitrogen | A-11008 |  | 1:1000 |
|  |  |  |  |  |  |
